# 2025 Update to the Female Athlete Triad Coalition Consensus Statement Part 1: State of the Science and Introduction of a New Adolescent Model

**DOI:** 10.1007/s40279-025-02333-z

**Published:** 2025-12-31

**Authors:** Mary Jane De Souza, Nancy I. Williams, Madhusmita Misra, Aurelia Nattiv, Elizabeth Joy, Michelle Barrack, Emily A. Ricker, Sasha Gorrell, Kristen J. Koltun, Emma O’Donnell, Rebecca J. Mallinson, Ana Carla C. Salamunes, Kary Woodruff, Michael Fredericson, Franziska Plessow

**Affiliations:** 1 Women’s Health and Exercise Laboratory, Department of Kinesiology, Pennsylvania State University, Noll Laboratory, University Park, PA 16802 USA; 2 Misra Laboratory, Division of Pediatric Endocrinology, Department of Pediatrics, University of Virginia, Charlottesville, VA USA; 3 Division of Sports Medicine, Department of Family Medicine, University of California Los Angeles, Los Angeles, CA USA; 4 Division of Sports Medicine, Department of Orthopaedic Surgery, University of California Los Angeles, Los Angeles, CA USA; 5 Lore Health, Salt Lake City, UT USA; 6 Department of Family and Consumer Sciences, California State University, Long Beach, Long Beach, CA USA; 7 Consortium for Health and Military Performance, Department of Military and Emergency Medicine, F. Edward Hébert School of Medicine, Uniformed Services University, Bethesda, MD USA; 8 Henry M. Jackson Foundation for the Advancement of Military Medicine, Inc., Bethesda, MD USA; 9 Department of Psychiatry and Behavioral Sciences, Weill Institute for Neurosciences, University of California San Francisco, San Francisco, CA USA; 10 Neuromuscular Research Laboratory, Department of Sports Medicine and Nutrition, University of Pittsburgh, Pittsburgh, PA USA; 11 School of Sport and Exercise Health Sciences, National Centre of Sports and Exercise Medicine, Loughborough University, Loughborough, Leicestershire UK; 12 Pennsylvania State University Harrisburg, Harrisburg, PA USA; 13 Nutrition and Integrative Physiology, University of Utah, Salt Lake City, UT USA; 14 Division of Physical Medicine & Rehabilitation, Department of Orthopaedic Surgery, Stanford University School of Medicine, Stanford, CA USA; 15 Stanford Prevention Research Center, Stanford University, Stanford, CA USA

## Abstract

This paper is the first of two publications comprising a 2025 update to the 2014 Consensus Statement on treatment and return-to-play guidelines on the Female Athlete Triad (Triad), defined as three inter-related components including energy status, reproductive function, and bone health. The Triad is initiated by exposure to varying degrees of energy deficiency with or without disordered eating/eating disorders with primary pathological outcomes to the reproductive and skeletal systems. This first paper includes a detailed update on the scientific underpinnings of the Triad and introduces a new Triad model specific to the adolescent female athlete. Energy deficiency and “metabolic compensation” are added to the energy status continuum to describe adaptations that reflect energy conservation. Ovarian steroid hormone exposure and functional hypothalamic oligo-amenorrhea are added to the reproductive function continuum. Bone stress injury is added to the bone health continuum. Rates of change are depicted for the induction and recovery of clinical outcomes within the adult model. Evidence-based statements are presented throughout the paper and supported by a high number of level A and B grades.

## Key Points

**Table:** 

In this 2025 updated Female Athlete Triad Coalition Consensus Statement, the term “energy deficiency” is used in association with the energy status spectrum instead of “low energy availability.” Low energy availability (defined as the difference between energy intake and exercise energy expenditure normalized for fat free mass) is now one of several indicators of energy deficiency in addition to low body weight and/or weight loss, low body mass index, and/or indications of metabolic compensation.
“Metabolic compensation” is introduced to refer to factors that reflect adaptive processes to reallocate the body’s energy resources for the purpose of energy conservation.
Bone stress injuries are added to the bone health continuum to reflect the well-established data demonstrating that Female Athlete Triad components are consistently associated with an increased risk of a bone stress injury, which often precipitates a stress fracture.
Ovarian steroid hormones have been added to the reproductive continuum in light of evidence demonstrating that nutritional interventions that provide increased energy intake not only result in the recovery of menses, but also the recovery of ovarian steroid concentrations and ovulation.
It remains questionable whether recovery of bone mineral density is possible following nutritional interventions of increased energy intake.

## Introduction

This paper is the first of two publications comprising a 2025 update to the 2014 Consensus Statement on treatment and return to play guidelines on the Female Athlete Triad (Triad) [1–3]. The Triad is defined as three inter-related components including energy status, reproductive function, and bone health [1–3]. The Triad is initiated by exposure to varying degrees of energy deficiency with or without disordered eating (DE)/eating disorders (EDs) with primary pathological outcomes to the reproductive and skeletal systems [1–3]. This first paper, *2025 Update to the Female Athlete Triad Coalition Consensus Statement Part 1: State of the Science and Introduction of a New Adolescent Model,* includes a detailed update on the scientific underpinnings of the Triad, introduces revisions to the current Triad model, introduces a new Triad model specific to the adolescent female athlete, and includes several emerging issues of importance in Triad science. The second paper, *2025 Update to the Female Athlete Triad Coalition Consensus Statement Part 2: Clinical Guidelines for Screening, Diagnosis, Treatment and Return to Play for Adolescents and Adults*, provides updated clinical information and a revised cumulative risk assessment tool for diagnosis, treatment, and return to play for female athletes. Both 2025 papers represent a consensus of the state of the science and recommendations for physicians, sports medicine practitioners, other healthcare providers, and researchers that was developed by the Female and Male Athlete Triad Coalition.

### The Consensus Process and Evidence Grading

An independent expert panel of 16 individuals with demonstrated expertise in the various topics related to Triad science and previous members of Triad consensus papers was convened to consolidate evidence-based data and develop recommendations. We drew on emerging data and the expertise of the diverse writing team of clinicians, sports dietitians, and scientists. The expertise of the group deemed necessary to properly address the current state of knowledge of Triad science included experts in the following subject areas: adolescent and adult EDs, psychology of eating behavior, biological and cognitive psychology, exercise physiology, cardiovascular physiology, kinesiology, nutrition, women’s health, pediatric and adult endocrinology, bone health, orthopedics, physical therapy, nutrition, reproductive medicine, and clinical sports medicine. No funding was involved in the recruitment or work of the panel. No conflicts of interest were declared.

A consensus was established using a strategy similar to that utilized by the 2014 Female Athlete Triad Coalition Consensus Statement [3]. The consensus process included one in-person group meeting, several virtual video meetings, and e-mail communications. The topics discussed in the Consensus Statement were divided amongst the writing team, and a leader was established for each section. Section leaders then worked with their teams to draft each section and develop evidence-based statements. Section leaders presented their team’s literature review findings, and a group discussion followed to develop a final consensus on the evidence-based statements. Each evidence-based statement underwent extensive discussion during video conferences. Exact language was developed, edited, and finalized. Grading of each statement was discussed and agreed upon only after a thorough group discussion. Next, online confidential voting on all evidence-based statements was used to determine consensus. Responses included “agree,” “neutral,” or “disagree.” If > 85% of writers “agreed” on a given evidence statement and there were no instances of “disagree,” no further discussion of the statement ensued. Discussion occurred if ≤ 85% of writers “agreed” and there were instances of “neutral” or “disagree.” Discussion resulted in either a revision of the statement or a deletion of the statement. Voting on revised statements was repeated with subsequent discussion and revision of statements, until all questions achieved > 85% of writers responding with “agree” with no instances of “disagree.” If recommendations from peer reviewers of the manuscript included revision of any evidence-based statements, the process of voting for those statements was repeated. The strength of the evidence-based statements was graded using a taxonomy in which randomized controlled trial (RCT) and observational data are considered the highest level of evidence, as utilized in the American College of Sports Medicine position stands and by the Agency for Healthcare Research and Quality [4, 5]. The specific evidence scoring criteria were:

Evidence Level A: Consistent pattern of findings based on substantial data from RCTs and/or observational studies.

Evidence Level B: Strong evidence from RCTs and/or observational studies, but with some inconsistent results from the overall conclusion.

Evidence Level C: Evidence from a smaller number of observational and/or uncontrolled or non-randomized trials, which is generally suggestive of an overall conclusion.

Evidence Level D: Insufficient evidence for categories A-C; panel consensus judgment.

The co-chairpersons (MJD and NIW) of the Consensus statement organized notetaking from each consensus meeting and compiled manuscript drafts. Drafts of the document were then circulated to the entire writing team for review and further editing ensued following each video conference until a final document was agreed upon after achieving a consensus by the entire writing team. The entire paper was reviewed by independent reviewers as per journal guidelines for *Sports Medicine*.

### What is the Value of a Consensus Statement Focused Specifically on the Female Athlete Triad?

Since the 2014 Triad Consensus Statement publication [2, 3], advances in scientific and clinical understandings of the Triad have progressed, necessitating an updated Consensus Statement summarizing the state of the science and recommended clinical practice. While no theoretical model of a medical condition is perfect, Triad researchers have always strived to present a model that is not only scientifically accurate, but useful for both clinicians and practitioners. Attention to the Triad is also warranted given the consistent increase in the number of girls and women participating in sport. For example, as of 2022, there has been a record number of female student-athletes competing in competitive sport worldwide [6]. Attention to the issue of the Triad has also grown as several high-profile athletes have spoken out regarding their Triad experiences on social media [7].

Last, the Triad is the foundation of the broadly described condition known as Relative Energy Deficiency in Sport (RED-S). Relative energy deficiency in sport was described in 2014 as a condition of impaired physiological and/or psychological functioning involving numerous physiological and performance outcomes experienced by female and male athletes and caused by exposure to low energy availability (EA) [8–10]. Initially, RED-S was framed as a more all-encompassing model than the Triad, inclusive of many more physiological endpoints that were purported to be causally tied to low EA in both female and male individuals. While the 2014 RED-S model includes the three components of the Triad, i.e., low EA with or without ED/DE, reproductive function, and bone health [10], a concern is that the RED-S model also includes several outcomes for which no RCTs are available to support its causal link to low EA, i.e., gastrointestinal and immune outcomes, and the model is applied to men despite the absence of the availability of data to describe causal links to low EA in men [11, 12]. Moreover, the clinical relevance of the Triad is obscured in the RED-S model because the manner in which it is depicted minimizes its relevance to simply a minor component of the RED-S model. However, in the 2023 RED-S update, Triad conditions are actually the major presentation of RED-S described as being caused by “problematic low EA” [8]. Specifically, the “primary indicators” of a RED-S diagnosis rendering the highest risk categorization, i.e., associated with “problematic EA,” are menstrual disturbances or low testosterone, low bone mineral density (BMD), or bone stress injuries (BSIs), and EDs. That said, the more recently published 2023 update of the IOC Consensus statement has since eliminated any mention of the Female or Male Athlete Triad and does not include clinically significant information specifically focused on Triad diagnosis, prevention, treatment, and return to play [8]. Despite this, the components of the Triad, i.e., EDs, reproductive disturbances, and low BMD/BSI still represent the RED-S outcomes with the highest prevalence and the largest and strongest body of evidence as to the causal link with energy deficiency. Thus, a diagnosis of RED-S, at its most severe presentation, continues to be based on diagnosing the presence of the Triad, as its components are the “most well-documented sequelae of problematic low EA” [8]. While research addressing the RED-S model is ongoing, the sustained relevance of its foundational origin, i.e., the Female and Male Athlete Triad, has been recognized by others [11–16]. Furthermore, recent publications have formally questioned the validity of the scientific evidence supporting the RED-S model [12, 16]. The latter publication describes the lack of supporting empirical scientific evidence and other concerns that are associated with the RED-S model, i.e., the low quality of evidence supporting the association between low EA and many of the RED-S outcomes and the likelihood that other factors, such as sleep, stress, and overtraining, may contribute significantly and independently to these outcomes. In summary, the sustained relevance and clinical importance of the Triad, when considered in the context of the less well scientifically defined concept known as RED-S, underscore the importance of this updated Triad Consensus Statement, which continues to evolve based on advances in the supporting science.

### Brief Review of the 1997, 2007, and 2014 Triad Position Stand/Consensus Statement Models

The Triad was first defined as the interrelated syndromes of DE, amenorrhea, and osteoporosis, the evidence for which was first published in 1997 [17]. As our scientific and clinical understanding of the syndrome evolved, updated position stands and consensus statements have been published in 2007 and 2014, which redefined the Triad as a continuum model [1–3, 18]. The 2014 Consensus Statement also detailed treatment and return-to-play guidelines and presenting a cumulative risk assessment tool for the Triad utilized the updated continuum model [1–3]. The 2025 current Consensus statement presents revisions to the 2007 continuum model based on advancements in the scientific and clinical understanding of the Triad to date.

### Presentation of the 2025 Update of the Adult Female Athlete Triad Model

**Fig. 1:**
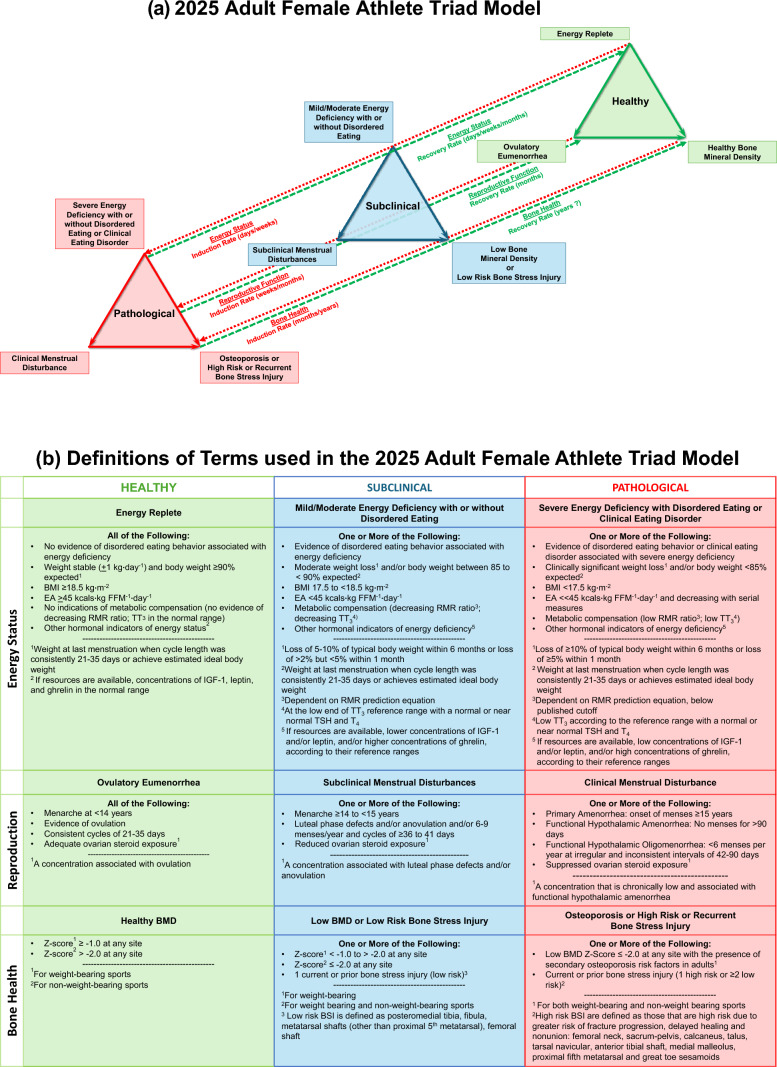
**(a)** The updated 2025 Adult Female Athlete Triad Model. The three inter-related components of the Female Athlete Triad are energy status, reproductive function and bone health. The Female Athlete Triad is initiated by exposure to varying degrees of energy deficiency with or without disordered eating/eating disorders with primary pathological outcomes to the reproductive and skeletal systems. Separate bi-directional arrows indicate the different rates of induction and recovery of each pathological outcome. The lack of data supporting the potential for complete recovery of bone health is noted by “?”. Chronic hypoestrogenemia resulting from energy deficiency induced reproductive suppression independently affects bone health. Updates include revised terminology describing energy status, i.e., energy deficiency instead of energy availability. Bone stress injury is now included in the bone health spectrum. *See Fig. 1b for an explanation of the detailed features of the Triad Continuum Components. **(b)** Definitions of terms used in the updated 2025 Adult Female Athlete Triad Model. Detailed descriptions of the terminology used to describe the healthy, subclinical, and pathological states of each Triad component have been revised according to recent research. *BMD* bone mineral density, *BSI* bone stress injury, *BMI* body mass index, *EA* energy availability, *IGF-1* insulin-like growth factor-1, *RMR* resting metabolic rate, *TSH* thyroid-stimulating hormone, *TT*_*3*_ total triiodothyronine, *T*_*4*_ thyroxine

As the expert panel considered the state of the science as of 2025, several updates to the Triad continuum model (Fig. 1a, b) were identified, as described below.

#### Energy Deficiency and “Metabolic Compensation” are Added to the Energy Status Continuum to Describe Adaptations That Reflect Energy Conservation

There is no validated or universally accepted approach to describe or quantify the body’s overall energy status or the specific partitioning of oxidative fuels to various physiological systems such as growth, reproduction, locomotion, or thermoregulation. As such, for the purposes of this Consensus Statement, we are defining terms relating to the energy continuum in the context of the scientific literature to date pertaining to the Triad where the specific focus is on understanding the metabolic status most aligned with the induction and reversal of Triad conditions. That said, advances in research support revised terminology describing both the concepts and quantitative metrics comprising the Triad energy status continuum. Notably, the term “energy deficiency” is now used in association with the energy status spectrum instead of “low EA.” Low EA (defined as the difference between energy intake and exercise energy expenditure normalized for fat-free mass [FFM]) is now one of several indicators of energy deficiency in addition to low body weight and/or weight loss, low body mass index (BMI), and/or indications of metabolic compensation. The rationale for this change is that EA is difficult to reliably estimate and can fluctuate widely from day to day [19]. Moreover, the calculation of EA alone is insufficient to accurately describe energetic status, as it does not capture body energy stores or physiological indications of metabolic compensation, a new term introduced in this revised Consensus Statement.

“Metabolic compensation” refers to factors that reflect adaptive processes to reallocate the body’s energy resources for the purpose of energy conservation. Indications of metabolic compensation are observed in response to the loss of body weight that may or may not represent a new chronically low, body weight set-point [20, 21] or low BMI [22, 23]. Metabolic compensation also includes the suppression of the resting metabolic rate (RMR) normalized for FFM [21], a reduced ratio of measured to predicted RMR, and/or sustained evidence of decreases in metabolic hormones, or levels that are low or at the lower end of the reference range, such as total triiodothyronine (TT_3_), insulin-like growth-factor-1 (IGF-1), leptin [24–26], and increased ghrelin [27, 28]. It is important to note that these hormonal and metabolic adaptations described as metabolic compensation are often associated with a state of weight stability, underscoring that repeated observations limited to measurements of body weight can be misleading. As well, body weight stability should not be interpreted as evidence of energy balance, as plasma volume expansion with training [29] and increased body water stored with training-induced increases in glycogen storage [30] can contribute to body weight increases and as such, may mask mild energy deficiency. Compensatory metabolic adaptations can occur over days to weeks in energy-deficient individuals and appear to be reversible, although less is known regarding the exact timeframes over which specific metabolic parameters return to levels associated with being energy replete [26, 31].

#### Bone Stress Injuries (BSIs) are Added to the Bone Health Continuum

The addition of BSI to the model reflects well-established data demonstrating that Triad components, i.e., menstrual disturbances, low body weight, and DE, are consistently associated with an increased risk of BSI, defined as the loads applied to bones that cannot be tolerated resulting in structural inflammation, fatigue, and local bone pain from repeated mechanical loads that can precipitate a stress fracture [32–34]. Stress fractures are defined as microdamage and resultant fractures that result from abnormal and repetitive loading in bone [35]. Notably, stress fracture rates in exercising women with functional hypothalamic oligo*-*amenorrhea (FHOA) are similarly high to those observed in military women, especially during basic combat training [36, 37]. As such, the subclinical range of the bone health continuum now encompasses “Low BMD or Low Risk BSI,” and the pathological range encompasses “Osteoporosis or High Risk or Recurrent BSI,” which are defined in Fig. 1b.

#### Ovarian Steroid Hormones, FHOA, and Bidirectional Lines Indicating Rates of Induction and Recovery are Added to the Reproductive Function Continuum

The addition of ovarian steroid hormones to the reproductive continuum is warranted in light of RCT evidence and other studies demonstrating the importance of nutritional interventions that provide increased energy intake for the recovery of not only menses, but also for the recovery of ovarian steroid concentrations and ovulation and the potential for downstream effects on bone [38–44]. Thus, even in the face of increased menstrual frequency, i.e., the resumption of a single menses after a period of amenorrhea, full recovery of ovarian steroids and ovulation is not always observed in studies involving nutritional recovery in female athletes and exercising women [39]. As such, resumption of menses, ovarian steroid concentrations, and the presence of ovulation are all of clinical significance and warrant consideration when defining the adequacy of reproductive recovery. Evidence from multiple RCTs also suggests that transitions along these menstrual-related spectra occur at different rates for induction and recovery, with induction occurring faster than recovery (see Fig. 1a) [25, 26, 38, 40, 42, 45, 46]. The addition of FHOA to the pathological end of the continuum demonstrates that care should be taken to rule out a hybrid-type presentation of oligomenorrhea associated with biochemical hyperandrogenism, as opposed to oligomenorrhea of hypothalamic origin, similar in etiology to functional hypothalamic amenorrhea [47, 48]. As such, we now refer to functional hypothalamic oligo-amenorrhea as FHOA.

#### Rates of Change are Depicted for the Induction and Recovery of Clinical Outcomes Within the Adult Model

Since the previous Triad Consensus Statement was published, much evidence has accumulated to demonstrate the rate of induction/recovery of changes in clinical outcomes, thus generating supporting refinements to the model [26, 38]. It is becoming evident that the rate of change for a particular outcome may be different if an individual is moving from “healthy” to “pathological” (induction), or from “pathological” to “healthy” (recovery). For instance, the REFUEL RCT demonstrated that within 12 months of refeeding, previously women with FHOA can have an increased frequency of menses [38]. In most participants who recovered menses, this increased frequency was noted in the first 3 months of refeeding. However, 12 months of refeeding was not long enough to re-establish regular ovulatory menstrual cycles or normal patterns of ovarian hormone exposure [39]. However, when recovery of menses was defined conservatively as two or three consecutive menstrual cycles of < 36 days, estrogen exposure is improved in these individuals in the REFUEL study [39, 49]. Notably, after 12 months of refeeding with a high proportion of participants resuming menses (defined as the occurrence of menses in amenorrheic participants or an increase in the number of menses in oligomenorrheic participants), BMD did not improve, perhaps indicating that the time frame over which refeeding needs to occur to improve bone health exceeds 12 months [50], or the volume of increased energy intake required to impact bone is greater, or that the energetic starting point, i.e., baseline body weight or body fat, or baseline RMR, or hormonal status of TT_3_ or IGF-1 may impact the time course of recovery. In another study with moderate caloric restriction and exercise training over 3 months, the induction of menstrual disturbances in previously ovulatory young, untrained women was associated with metabolic changes such as weight loss, reductions in RMR, decreases in TT_3_, IGF-1, and leptin and increases in ghrelin [26]. The magnitude of the energy deficit was proportional to the frequency of observed menstrual disturbances, all within 3 months [26]. In the updated model figure, rates of change are noted along “Induction Rate” and “Recovery Rate” lines.

#### A Question Mark (?) Indicates the Uncertainty of Bone Mineral Density (BMD) Recovery, Which Requires Clarification Through a Stronger Evidence Base

It is questionable whether recovery of BMD is possible following an increased energy intake. After a 12-month intervention (REFUEL RCT) of approximately 350 kcal/day in exercising women with FHOA, BMD did not improve at any site despite increased weight and fat mass compared to the control group [50]. A 20-week intervention that increased the energy intake by 360 kcal/day and reduced the training load by 1 day/week resulted in significant improvements in lumbar spine and femoral neck BMD in a small group (*n* = 7) of amenorrheic athletes [51]. However, that study did not include a control group and the complete study methods were not described [51]. A longer duration of increased energy intake, a greater magnitude of energy intake, specificity of varying macronutrient composition, and consideration of baseline demographics may impact the potential for improvement in BMD.

#### New Details Focused on the Adolescent and Accompanying Model Figure Are Introduced

**Fig. 2:**
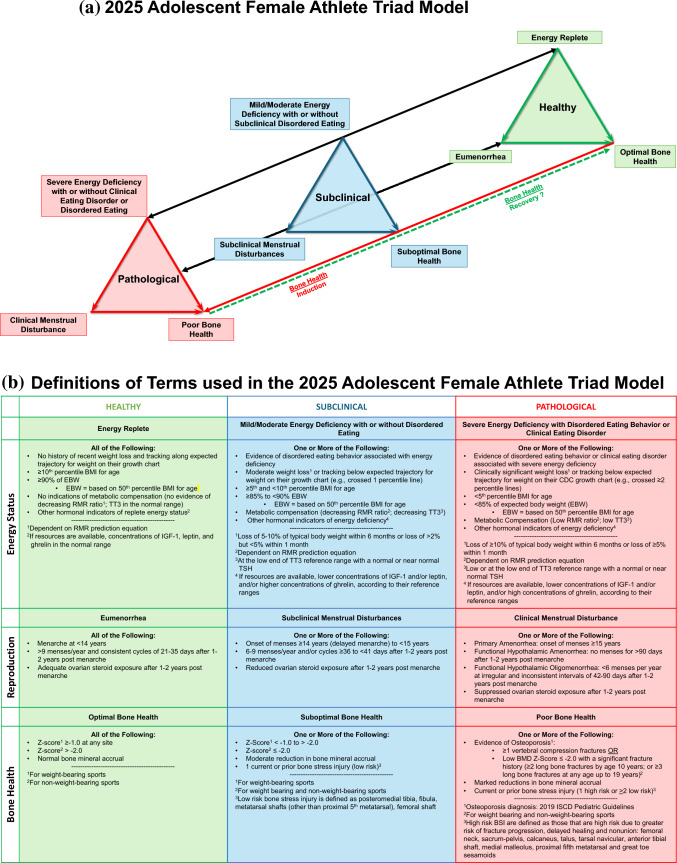
**(a)** The new 2025 Adolescent Female Athlete Triad Model. The three inter-related components of the Female Athlete Triad are energy status, reproductive function and bone health. The adolescent Female Athlete Triad is initiated by exposure to varying degrees of energy deficiency with or without disordered eating/eating disorders with primary pathological outcomes to the reproductive and skeletal systems. Chronic hypoestrogenemia resulting from energy deficiency induced reproductive suppression independently affects bone health. Bi-directional arrows indicate both induction and recovery of the pathological outcomes for energy status and reproductive status. Separate arrows and “?” for bone health indicate the lack of data supporting the potential for complete recovery of bone health. *See Fig. 2b for an explanation of the detailed features of the Triad Continuum Components. **(b)** Definitions of terms used in the new 2025 Adolescent Female Athlete Triad Model. Detailed descriptions of the terminology used to describe the healthy, subclinical, and pathological states of each Triad component have been revised according to recent research. *BMD* bone mineral density, *BSI* bone stress injury, *BMI* body mass index, *EA* energy availability, *EBW* expected body weight, *IGF-1* insulin-like growth factor-1, *ISCD* International Society for Bone Densitometry, *RMR* resting metabolic rate, *TSH* thyroid-stimulating hormone, *TT*_*3*_ total triiodothyronine

Female adolescent athletes may be more severely impacted by the Triad given potential disruptions to bone mineral accrual and the risk of developing a low peak BMD, which underscores the need for early identification, treatment, and prevention efforts. Energy deficiency in adolescent female athletes can result in delayed menarche or primary amenorrhea, secondary amenorrhea, low BMD, reduced bone accrual, and low peak bone mass [52–57]. An adolescent Triad model is introduced in this update (Fig. 2).

## Scientific Updates to the Energy Status Continuum

### General Comments and Definitions of Energy Terminology

**Fig. 3:**
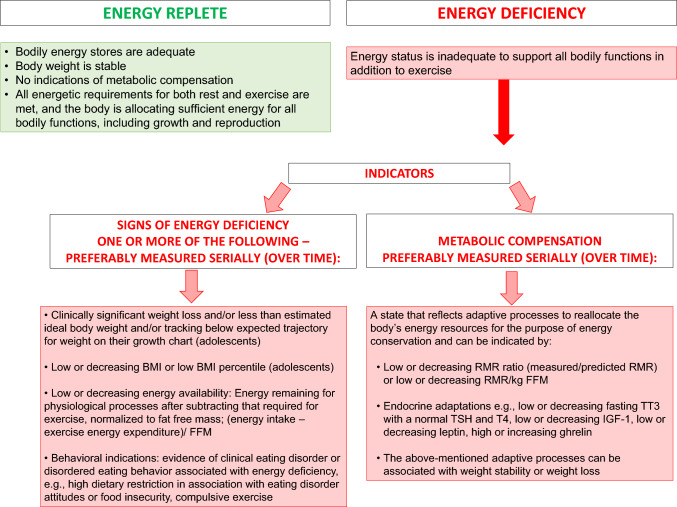
Definitions for revised terminology for the energetic components of the 2025 Adult and Adolescent Female Athlete Triad Models. *BMI* body mass index, *FFM* fat free mass, *IGF-1* insulin-like growth factor-1, *RMR* resting metabolic rate, *TSH* thyroid-stimulating hormone, *TT*_*3*_ total triiodothyronine, *T*_*4*_ thyroxine

Describing the causal role of energy status in the induction and reversal of Triad outcomes requires clear and consistent use of terminology. We offer the following definitions and advocate for consistent use in all future Triad literature. We provide definitions below, where each is discussed, and in Fig. 3, for ease of reference.

#### Energy Replete

*Energy replete* refers to a state where bodily energy stores are adequate, circulating fuels and glycogen stores are adequate, body weight is stable or increasing, and there are no indications of metabolic compensation (described in Sect. 2.1.4.). In an energy replete state, all energetic requirements for both rest and exercise are met, and the body is allocating sufficient energy for all bodily functions, including growth and reproduction.

#### Energy Deficiency

**Fig. 4:**
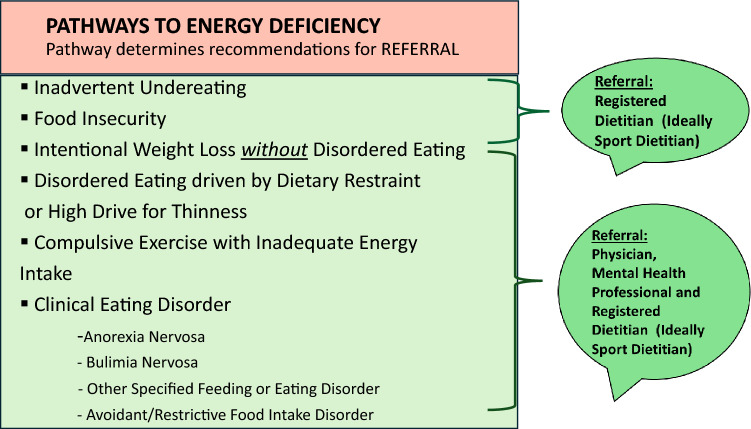
Pathways to energy deficiency along with referral recommendations

*Energy deficiency* occurs when energy status is inadequate to support all bodily functions in addition to exercise and can be identified by the presence of one or more of the following: recent weight loss, low or decreasing BMI or low BMI percentile (adolescents), low or decreasing EA, as defined below, and/or indications of metabolic compensation, as defined below. Behaviorally, energy deficiency can develop through many pathways, i.e., clinical ED, DE, intentional weight loss without DE, inadvertent undereating, and maladaptive exercise behavior (e.g., compulsive exercise), as shown in Fig. 4. Compulsive exercise is only one of several terms that refer to exercise behavior intended to control weight or shape or to regulate ED-related emotions that leads to impairments in functioning [58, 59]. This symptom is not rare; it may occur across all EDs (including those associated with binge eating and/or overweight [60], and among a majority of those with binge-spectrum EDs (79%) [58, 60–63] and anorexia nervosa [80%]). Most notably, across studies, compulsive exercise contributes to an increased suicide risk [64], poorer treatment outcomes [61], lower quality of life [65], and an increased risk for relapse [66]. With chronic energy deficiency (weeks-months), the physiological cascade of Triad-related conditions can progress.

#### Energy Availability (EA)

Energy availability refers to the amount of dietary EA to sustain physiological function after subtracting the energetic cost of exercise [67]. EA is quantitatively calculated by subtracting the kcals expended during exercise (not including RMR) from the kcals of a 24-h dietary intake, and normalizing the difference for FFM [68]:

$${\text{Energy availability}} = {\text{energy intake}} \left( {{\mathrm{kcals}}} \right) - {\text{exercise energy expenditure}} \left( {{\mathrm{kcals}}} \right)/{\mathrm{fat}} - {\text{free mass}} \left( {{\mathrm{kg}}} \right).$$

An EA of approximately 45 kcal/kg FFM/day has been associated with the healthy end of the Triad continuum, i.e., being energy replete, having ovulatory eumenorrheic menstrual cycles, and having normal BMD. Whether an EA < 30 kcal/kg FFM/day represents a threshold below which Triad-related physiological changes begin to occur has been debated, and in this revised Consensus Statement, the utility of an EA threshold is less supported than in previous position stands/consensus statements [69, 70].

#### Metabolic Compensation

*Metabolic compensation* as a “concept” refers to a state that reflects adaptive processes to reallocate the body’s energy resources for the purpose of energy conservation. Quantitative indicators of metabolic compensation include a ratio of measured to predicted RMR that is decreasing or below reported cutoffs (i.e., 0.90 if using the Cunningham_1980_ or Harris-Benedict prediction equations, 0.92 if using the Cunningham_1991_ prediction equation, or 0.94 with dual-energy x-ray absorptiometry-predicted RMR) [71], or reduced RMR/kg FFM or TT_3_ concentration that is low with normal or near-normal thyroid-stimulating hormone and thyroxine concentrations [72–74]. Other adaptations include attempts to reallocate energy away from growth (low or decreasing IGF-1), to reduce satiety (low or decreasing leptin), or to increase hunger (high or increasing ghrelin) [75–77].

### Is There a Threshold of EA that is Associated with Menstrual Disturbances?

Since the publication of the 2007 Female Athlete Triad position stand [18] that highlighted the key role of low EA as a causative factor in the etiology of the Triad, an EA threshold below which menstrual disturbances occur has been a topic of inquiry. Notably, since Loucks et al. [24, 68] observed reductions in 24-h measured luteinizing hormone (LH) pulse frequency after 4–5 days of EA maintained below a threshold of 30 kcal/kg FFM/day, it has been proposed that menstrual disturbances would be induced below this threshold.

Randomized controlled trial evidence demonstrated that an energy deficit of − 22% to − 42% of baseline energy needs (− 470 kcal to − 810 kcal below initial energy requirements) imposed via energy restriction and exercise energy expenditure for three menstrual cycles induced luteal phase defects, oligomenorrhea, and anovulation [26], supporting earlier prospective investigations by Bullen et al. [45, 46] and establishing actual estimates of the magnitude of energy deficit associated with reproductive perturbation. Further, a secondary analysis demonstrated that in untrained women initiating an exercise program, EA was linearly related to the risk of menstrual disturbances, but no clear threshold existed below which ovarian function was disrupted [69]. That is, many women with ovulatory cycles had EA levels below 30 kcal/kg FFM/day, and women with menstrual disturbances had EA levels above 30 kcal/kg FFM/day [69]. Nonetheless, as EA dropped below 30 kcal/kg FFM/day, the risk of a menstrual disturbance increased by more than 50% [69]. Independent of the EA value below which menstrual cycles may be disturbed, a recommendation to strive for an EA value of approximately 45 kcal/kg FFM/day is supported. Additional research addressing energy and/or metabolic thresholds is necessary.

**Evidence-Based Statement:** Research in untrained women who underwent a 3-month exercise intervention that included caloric restriction does not support the use of an absolute threshold of EA as a strategy to prevent menstrual disturbances; rather, there is individual variability in the level of EA below which menstrual disturbances are induced and there likely exists a dose–response continuum between energy status and menstrual function. **Grade:** Level A.

### What are the Measurement Limitations of Assessing EA?

Although EA has historically been the primary metric of energetic status within the Triad model, much has been written to describe its limitations, and we refer the reader to those publications for a full discussion on this issue [78, 79]. In short, it is well established that EA is difficult to measure in the laboratory [78] and especially in the field, particularly given the dependence on the self-report of energy intake and the complexity of accurately assessing exercise energy expenditure [80].

Beyond calculation methods, an additional concern regarding measures of EA is the timeframe reflected and whether it aligns with the physiological signs of metabolic compensation that underlie Triad sequelae. For example, EA may have wide variations across days, weeks, and months, and depending on when it is assessed, EA may not align with the timing of physiological adaptations. Typically, EA has been assessed during a single day (e.g., in 24-h blocks); however, there has been growing interest in investigating different timeframes during which EA can fluctuate.

Fluctuations in energetic status over shorter timeframes, i.e., hourly, may also be associated with physiological adaptations to energy deficiency [81–84]. As such, recent attention has been directed to a “within-day energy balance” assessment. Although individuals may have adequate energy status on a daily basis, there can be deficits during specific periods of the day, reaching as much as 1000 kcal/h, or with smaller scale deficits of > 300 kcal/h occurring for as many as 6 h of the day [82], which may contribute to metabolic compensation. As such, more research on the importance of assessments of energy status over different time periods may help elucidate the temporality of compensatory metabolic changes in response to low EA.

Because of concerns regarding the variability of EA assessments, it is proposed that EA measurements be used in conjunction with other quantitative measurements of energy status to assess an at-risk profile for Triad sequelae. Measurements that can be serially monitored across time in the same free-living individual, such as body weight and composition, RMR, the ratio of measured to predicted RMR ratio, TT_3_, sensor-based physical activity monitoring, and measures of restrictive and/or undereating, may be more informative and predictive of the initial induction of downstream Triad conditions than adopting a particular absolute value of EA that was derived from short-term laboratory-based studies in non-exercising eumenorrheic women. Going forward, efforts should be made to develop specific recommendations for when and how to use measures of energy status and to validate field- or wearable-based approaches to determine athletes’ energy status. Measures that are easily obtained in clinical and field settings, i.e., body weight, BMI, BMI percentile, BMI expressed as a percentage of expected, wearable technology metrics, and perhaps measures of eating behavior and body composition should be prioritized when other resources are not available.

**Evidence-Based Statement:** In non-laboratory settings, the measurement of EA is problematic because of low reliability in the assessment of its components and high day-to-day fluctuations***.* Grade:** Level B.

## Scientific Updates on Eating Disorders (EDs) and Disordered Eating (DE)

### General Comments

Disordered eating is defined by the Academy of Nutrition and Dietetics as “a range of irregular eating behaviors that may or may not warrant a diagnosis of a specific ED” [85]. Eating disorders are psychiatric conditions characterized by severe and persistent disturbance in eating behaviors and associated distressing thoughts and emotions [86, 87]. Eating disorder classifications that are most likely to impact athletes include anorexia nervosa, bulimia nervosa, binge ED, and atypical anorexia nervosa, a variant of other specified feeding and other specified ED [87]. Athletes are at greater risk for developing DE and ED, a vulnerability that typically increases with higher levels of competition and as athletes face specific pressures to optimize their body composition, shape, and weight to perform [88–90]. Other psychological factors such as anxiety and depression can also impact pathways to clinical EDs and DE behaviors [91, 92]. Though not all athletes with the Triad exhibit pathological eating attitudes or behaviors, energy deficiency may result from DE or an ED.

For decades, researchers have sought to better understand the relationship between sport and ED: how best to identify athletes with DE behaviors or ED; underlying causes of EDs in athletes; management of ED in athletes, including return to play; and perhaps most importantly, prevention of ED in athletes [90]. It is essential that members of the athlete care team (i.e., physicians, athletic trainers, sports dietitians) recognize athletes with DE and ED and intervene early to prevent adverse health consequences [90].

### What Factors Increase the Risk for DE and EDs Among Athletes?

As noted in Sect. 3.1, almost 90% of athletes participating in a recent survey endorsed DE behaviors [93], which are likely to result from a variety of different predisposing factors. Over and above what is known of the causes of EDs in the general population, athletes exist within environments that can exacerbate their own individual vulnerability. In the following text, we describe both sport- and athlete-specific and athlete-contextual factors that may contribute to an increased risk for engagement in DE behaviors. Many athlete-specific factors interact with or are potentiated by contextual factors; we highlight notable examples as they are presented.

#### Sport-Specific Factors that Might Contribute to EDs and/or DE

Table 1 lists sport-specific factors that contribute to the etiology of ED and DE behaviors in athletes. For some individuals, athletic participation can be protective against body dissatisfaction, with some research reporting no differences between adolescent female figure skaters and non-skaters [94], or even improved body satisfaction among female collegiate athletes compared with non-athletes [95]. However, most of the research continues to support the specific contribution of sport type to the development of body dissatisfaction and related DE behaviors. The three-sport types most often identified with a heightened risk for DE behaviors are (1) leanness sports that emphasize a thin physique for perceived performance benefits, such as distance running [96]; (2) aesthetic sports where athletes are evaluated based on appearance such as dance [97]; and (3) weight-class sports where weight criteria are stipulated such as rowing [98]. Of note, a recent meta-analysis (*n* = 56 studies of female athletes across sport types) initially found that mean differences between athletes and non-athletes were non-significant for self-reported loss-of-control eating, dietary restriction, and drive for thinness; however, further analysis revealed sport type as a significant moderator of body dissatisfaction differences between athletes and non-athletes, such that larger effect sizes were observed in studies involving aesthetic or leanness sports, and athletes participating in aesthetic/leanness sports reported more ED pathology compared with athletes participating in non-aesthetic/non-leanness sports [99].

**Table 1:** Risk Factors for Eating Disorders and Disordered Eating in Athletes

Type	Notes
*Sport specific*	
Early sport specialization	Initiation of early sport-specific training, particularly in sports with idealized body types (e.g., aesthetic sports)
Sport type	Weight-class sports, aesthetics sports, sport positions (e.g., cheerleading base vs flyer), endurance sports, and sports in which athletes compete in revealing clothing
Athlete identity	Degree to which one identifies with the athlete role
Coach/teammate factors	A coaching style critical of body weight and composition; fear of judgment or non-selection by coaches or teammates’ influence; participation in weight-regulation practices (i.e., weigh-ins or frequent body composition assessments)
Trauma	Experiencing a traumatic event such as an injury; retirement from sport
*General*	
Individual factors Trauma and mental health disorders [126]	Perfectionism, (over-) compliance, high-achievement orientation, or obsessive–compulsive tendencies; orthorexia or obsessive preoccupation with healthy eating; driven or compulsive exercise Anxiety; depression; substance use disorder; obsessive compulsive disorder, post-traumatic stress disorder; abuse; sexual violence; borderline personality disorder, obsessive–compulsive personality disorder
Psychosocial factors [130]	School/academic; relationships (family, friends, teammates/coaches); societal pressures (pressure to be thin); financial (food insecurity); stress; social media engagement
COVID-19 [125]	Significant increase in eating pathology has been observed (in athletes and non-athletes) through the COVID-19 pandemic

*COVID-19* coronavirus disease 2019

Second, research suggests that early sport specification (i.e., striving for a sport-specific body ideal from a young and/or pre-pubertal age) may contribute to DE, along with the possibility that body ideals and the associated risk for DE may vary relative to sport position (e.g., flyers vs base for cheerleading) [100, 101]. Additionally, although research in this domain is more limited, athlete identity, namely the degree to which one identifies with the athlete role, may moderate differences in engagement in DE behaviors both in elite and non-elite athletes [94, 102, 103] and may be particularly salient in contributing to ED development for athletes who aspire to a certain body type, such as within ballet [104].

While physical activity is widely considered to be protective against negative physical and mental health outcomes, across demographic groups [105], athletes exist within a culture where the influences of coaches and teammates may serve as both risk and protective factors for eating pathology [106, 107]. Research on the systemic impact of coach and teammate influence is minimal, but current evidence suggests that DE behaviors may be positively reinforced by coaches, peers, and family, which can hinder acknowledgment of any ED-related issues and reduce help-seeking [93, 108]. In addition, some high-performance athletic contexts promote a culture of “mental toughness” in which fear of judgment or non-selection by coaches or teammates can inhibit help-seeking for ED, particularly for elite athletes [109]. Elite status continues to be a consistent contributor to an increased risk for DE behaviors, as evidenced in recent work comparing lower level versus more elite adult and adolescent female athletes [110, 111]. As noted above, it is also possible that traumatic events, such as an injury, and/or retirement from sport may negatively impact mental health.

#### General Factors that Might Contribute to EDs and/or DE

Table 1 lists additional general non-sport-specific factors that also contribute to the risk of ED and DE behaviors in athletes. Individual factors, such as perfectionism or achievement orientation, can contribute (adaptively) to greater athleticism and augment (maladaptively) adherence to strict dietary or exercise regimes [112, 113]. Orthorexia, or an obsessive preoccupation with healthy eating, does not constitute a formal ED diagnosis; however, it can contribute to engagement in DE behaviors [114, 115]. For athletes invested in achieving a certain body type or athletic milestone, efforts to eat “healthy,” “clean,” or lose weight based on the belief that it will be performance enhancing may be more common [116].

Over-exercising can be culturally sanctioned or rewarded depending on a given athletic culture, and even when ED-related symptoms are observable among athletes, they may be misinterpreted as performance enhancing when perceived within a sport environment [117]. In the context of eating pathology, early descriptions of problematic exercise focused on the “excessive” nature of this behavior, measured by time and/or intensity [118]. Recent evidence suggests that over and above the amount of exercise, *motivation* for exercise behavior (e.g., weight loss) reinforces rigidity in exercise routines (e.g., inability to cease activity, even when injured) [119] and is highly predictive of DE behaviors [120]. In the context of eating pathology, compulsive exercise is generally defined as driven physical activity that is undertaken to avoid a negative affect (e.g., guilt or anxiety) [121] and/or in the interest of weight/shape control [119]. Athletes engage in more compulsive exercise than non-athletes (11% vs 5.5%, respectively) [93], although some research suggests these numbers may be even higher, given that athletes may underreport this behavior [122]. High training demands and intensive workout schedules can make it challenging to determine whether any activity undertaken is over and above what is appropriate for a given athlete, and athlete identity promotes the likelihood of compulsive exercise behavior independent of the presence of eating pathology [123]. While more research in this area is warranted, taken together, it appears that energy deficiency likely occurs secondarily to compulsive exercise behavior among athletes, both generally, and more specifically in intersection with ED pathology [124].

Athletes cannot escape environmental influences such as the coronavirus disease 2019 pandemic [125], or the effects of trauma related to life events [126, 127]. Apart from sport-related pressures to be thin, societal expectations for the ideal body can exert pressures in addition to other non-athlete-specific stressors, such as relationships with family and friends, and financial or food insecurity [128, 129]. Individual susceptibility to these environmental influences can be modulated by factors such as a genetic predisposition to ED and mental health disorders (e.g., anxiety, depression, substance use disorder, obsessive–compulsive disorder, post-traumatic stress disorder) [Table 1] [91, 92].

**Evidence-Based Statement:** Although some aspects of sport participation are protective against negative mental health outcomes, female athletes may be at a unique risk for the development of eating pathology, particularly when engaged in sports that promote a certain body type. **Grade**: Level B.

**Evidence-Based Statement:** ED risk factors for female athletes include sport-specific (such as early sport specialization and coach/teammate influence) and general factors (including perfectionistic temperament and stress). **Grade:** Level C.

### What are the Health Consequences of EDs and DE in Athletes?

The health consequences of ED are numerous, including impairments to both mental and physical health. Although the Triad defines the relationship between energy deficiency (with or without DE or an ED), menstrual disturbances, and low BMD and/or BSI, EDs affect nearly every system in the body, adversely impacting health, ability to function, quality of life, and sport performance [90].

Regarding negative mental health sequelae, EDs are often accompanied by comorbid psychiatric conditions, the most common of which are anxiety (comorbidity rates up to 62%), mood (up to 54%), and substance use and post-traumatic stress disorders (rates up to 27%) [130]. Studies that have reported specifically on the mental health of female athletes have highlighted similar mental health concerns as those evidenced in the general population [131] along with the possibility that the risk for eating and associated psychopathology might be exacerbated by elite status [132] (e.g., among ultramarathon runners [131]). Studies examining associations among DE behaviors, amenorrhea, and estrogen status in athletes have identified higher depression and anxiety symptomatology among those with amenorrhea compared with those without amenorrhea, with amenorrheic athletes demonstrating greater DE [133, 134], reflecting the complex interplay between psychological distress and DE, and highlighting the need for comprehensive mental health support for these coexisting conditions [135, 136].

The wide-ranging physical health consequences of various DE behaviors are also well documented, and include broad negative clinical impacts on cardiovascular, gastrointestinal, and cognitive outcomes, among others (see Hambleton et al. [130] for a review) [Table 2]. Specific to female athletes, the disruption of normal endocrinological function in the context of DE and/or the Triad [137] also contributes to musculoskeletal effects, potentially resulting in low BMD, BSI [138–140] and may lead to growth restriction, particularly if the individual begins intense athletic activity prior to puberty [141, 142]. It is interesting to note that in military women, EDs/DEs are associated with BSI and musculoskeletal injury, although the mechanism may be related to poor sleep rather than energy deficiency [143]. Physicians' and other stakeholders’ awareness of these adverse mental and physical health effects is critical for early identification and timely intervention [90].

**Table 2:** Physical Health Consequences of Eating Disorders

Physical health	Behavior(s)	Signs/symptoms
Ear, nose, and throat/dental	Self-induced vomiting	Dental caries Recurrent sore throat
Cardiovascular	Dietary restriction, purging	Hypotension Orthostatic hypotension Syncope Sinus bradycardia Tachycardia Decreased left ventricular mass Increased left ventricular sphericity index Diastolic dysfunction Decreased cardiac output Decreased stroke volume Pericardial effusions Myocardial fibrosis Mitral valve prolapse Atrial ventricular conduction delay QTc wave prolongation Arrhythmia Autonomic dysfunction/heart rate variability changes Dysregulation of peripheral vasoconstriction/vasodilatation Arterial vasospasm Hypokalemia
Gastrointestinal	Dietary restriction Purging (can include compensatory behaviors such as laxative misuse, exercise, diuretics, medication misuse)	Gastroesophageal reflux disease Bloating Abdominal pain Gastroparesis Constipation Diarrhea Rumination
Musculoskeletal	Dietary restriction Purging	Low bone density, impaired bone geometry and microarchitecture, reduced strength estimates Reduced bone accrual in adolescents Bone stress injury Growth restriction (when athletic activity sufficient to cause the Triad starts before the age of 12–13 years) Delayed healing
Endocrinologic	Dietary restriction Purging	Reduced or suppressed secretion of gonadal steroids (secondary to functional hypogonadotropic hypogonadism) Reduced secretion of total T3 (to the lower end of the normal range, or sometimes frankly low) with normal or near normal TSH and T4 concentration Reduced secretion of IGF-1 Increased secretion of cortisol Altered secretion of hormones impacting appetite: Reduced secretion of leptin, oxytocin, insulin Increased secretion of ghrelin and PYY Reduced secretion of irisin and FGF21
Reproductive	Dietary restriction Purging Binge eating	Delayed menarche Primary amenorrhea Secondary amenorrhea or oligomenorrhea (functional hypothalamic oligo/amenorrhea) Infertility Complications of pregnancy
Neurologic	Dietary restriction Purging	Neuropathy (vitamin deficiencies) Possible cognitive impairment (verbal memory and executive function)

*FGF21* fibroblast growth factor 21, *IGF-1* insulin-like growth factor-1, *PYY* peptide YY, *T4* thyroxine, *total T3* total triiodothyronine, *TSH* thyroid-stimulating hormone

## Scientific Updates to the Reproductive Function Continuum

### General Comments

Without the necessary energy intake to meet energy expenditure needs, metabolic compensation ensues and redirects available energy away from reproduction [144]. The outcome is menstrual disturbances, which range from subclinical, such as luteal phase defects and anovulation, to pathological, including FHOA [1–3, 26, 45, 46, 145]. Multiple neuromodulatory pathways play a role in the origin of menstrual disturbances [48]. In short, reduced feedforward signaling of gonadotropin-releasing hormone results in altered LH pulse dynamics [24, 76, 146], compromised folliculogenesis and ovulation [147], and resultant hypogonadotropic hypoestrogenism. Most studies published to date support the notion that amenorrheic exercising women have intact pituitary responsiveness to graded doses of gonadotropin-releasing hormone [147–151].

Key research advances since the previous Female Athlete Triad Consensus Statement include (1) more specificity regarding the association between measurable indicators of EA and/or chronic energy status and menstrual dysfunction, (2) better understanding of the effects of hyperandrogenism on menstrual disturbances in exercising women, (3) insights regarding factors that influence the susceptibility of the hypothalamic-pituitary-ovarian (HPO) axis to energy deficiency, such as gynecological age and psychosocial factors, and (4) advances in the understanding of reproductive recovery in response to increased caloric intake.

### What is the Relationship of Energy Deficiency and Weight Loss to Menstrual Disturbances?

Prospective research findings demonstrate that menstrual disturbances can be induced in young women (aged 18–30 years) with moderate weight loss that ranged from 3.1 kg to 4.0 kg, throughout 1–3 months of energy restriction [26, 45, 46]. In contrast, 4 months of energy restriction in exercising women who lost approximately 3–9 kg but who were older, i.e., 25–40 years, produced declines in estrogen and progesterone exposure, but no changes in menstrual cyclicity [152]. In younger ovulatory exercising women, the volume of energy deficit associated with the initial onset of menstrual disruption ranged from 470 to 810 kcal/day [26]. The onset of luteal phase defects is the most frequently observed disruption, occurring at a rate that is directly related to the magnitude of energy deficiency [26]. The prospective induction of energy deficiency across a 3-month period resulted in a 23% reduction in LH pulse frequency, and every 0.1-unit decrease in LH pulse frequency was associated with 22 times greater odds of luteal phase defects than having an optimal ovulatory cycle [153]. Menstrual disturbances, especially when severe (i.e., FHOA), are associated with chronic suppression of ovarian steroid concentrations, including estrogen and progesterone, to levels comparable to those observed in postmenopausal women [154]. In exercising women with luteal phase defects and anovulation, concentrations of estradiol and progesterone are also suppressed, but not to the severity observed in women with FHOA [154].

**Evidence-Based Statement:** Menstrual disturbances associated with reduced or suppressed ovarian steroid exposure are induced in young normal weight, ovulatory women with moderate weight loss of 3–4 kg and with a moderate energy deficit of 500–800 kcal/day over 2–3 months. **Grade:** Level A.

Regarding other signs of metabolic compensation, female athletes with ED/DE and energy-related FHOA may exhibit low or low normal TT_3_ or free T_3_ levels while exhibiting normal or near normal thyroid-stimulating hormone and thyroxine levels [73, 74, 155–158]. With energy repletion, increasing energy, and/or resumption of menses, the TT_3_ level may normalize or approach normal values, and can be used as a surrogate marker for improved energy status [156].

**Evidence-Based Statement:** Total or free T_3_ can be used as a surrogate marker for energy status in female athletes with energy deficiency and resulting reproductive sequelae, and concentrations often normalize with improved energy status. **Grade:** Level B.

### How does Hyperandrogenism Complicate the Study of Menstrual Disturbances in Exercising Women?

In some exercising women, the cause of menstrual disturbances may be related to hyperandrogenism (elevated free androgen index [FAI]) rather than hypothalamic inhibition secondary to energy deficiency [47, 159, 160]. Conditions of hyperandrogenism, such as polycystic ovary syndrome (PCOS) and non-classic congenital adrenal hyperplasia often present with menstrual disturbances, including FHOA [161–163]. Distinguishing between underlying causes of FHOA can be challenging in the face of hyperandrogenism, as symptoms beyond menstrual disturbances are not always overt. For example, the diagnosis of PCOS, the most common endocrine disorder in women of reproductive age, is based on the presence of clinical or biochemical hyperandrogenism, oligo-anovulation, and/or multi-cystic ovaries. The combination and severity of symptoms can vary, leading to heterogeneous presentations that can complicate the diagnosis, particularly in women without overt clinical signs of hyperandrogenism [47, 159, 160].

In some exercising women, FHOA may co-occur with hyperandrogenism [160]. Koltun et al. reported that 17% of 100 women with FHOA recruited to participate in research studies also had hyperandrogenism, as evidenced by an FAI > 4.26 (FAI = total testosterone/sex hormone binding globulin)*100) [47]. As such, clinicians and researchers investigating women with FHOA should consider the androgen status in addition to other factors contributing to FHOA, recognizing that the etiology of menstrual disruption may be multi-faceted. Further, it is important to consider the unique phenotypic presentation of hyperandrogenism (i.e., higher BMI and body fat) when compared to exercising women with FHOA in order to determine whether the underlying cause of the menstrual disturbance is hyperandrogenism, energy deficiency, or both.

In “hybrid” cases, when both energy deficiency and hyperandrogenism are present, the hypothalamic inhibition of the HPO axis secondary to energy deficiency may suppress the typical symptoms of hyperandrogenism and mask symptoms characteristic of PCOS. In the aforementioned sample of exercising women with FHOA with a high FAI, clinical symptoms of hyperandrogenism were not apparent (e.g., hirsutism, acne) [47]. In fact, some have suggested that as many as half of all patients with PCOS and a non-hyperandrogenic phenotype (namely oligomenorrhea and polycystic ovarian morphology on ultrasound) may also have hypothalamic inhibition of the HPO axis [48]. Likewise, based on the possibility of existing hyperandrogenism in some exercising women and the possibility that hyperandrogenism may coexist with hypothalamic inhibition, special care must be taken when evaluating women with FHOA to consider androgen excess. Another consideration is the increased activity of the hypothalamic–pituitary–adrenal axis in FHOA [75, 164, 165], and whether this increased activity could result in an over-secretion of adrenal androgens, mimicking biochemical features of PCOS.

### How Does Gynecological Age Affect the Induction of Menstrual Disturbances?

Older gynecological age (defined as the difference between chronological age and age of menarche), has been reported to reduce susceptibility to energy deficiency-related menstrual disturbances, yet this finding has not been well addressed in the literature [166]. Gynecological age is lower in individuals in whom menarche is delayed but is equally influenced by chronological age. Evidence that gynecological age modifies the effects of energy deficiency on LH pulsatility was reported by Loucks [167], who demonstrated that women with a younger gynecological age (5–8 years) experienced a decrease in LH pulse frequency in response to short-term low EA under experimental conditions, while a decrease was not observed in women of more advanced gynecological age (14–18 years). Williams et al. [152] observed few disruptions in menstrual cycle regularity in normal-weight women (age 25–40 years, gynecological age of 11–29 years) who participated in a 4-month weight loss program consisting of exercise training combined with caloric restriction, in which weight loss exceeded that observed in another prospective study of younger women (age 18–30 years, average gynecological age ~ 8 years), in whom menstrual disturbances were observed in response to an induced energy deficit [26]. Taken together, data indicate that the risk of developing menstrual disturbances in association with energy deficiency declines with advancing gynecological age (independent of age of menarche). This is an important translational finding as clinicians should consider the gynecological maturity of an athlete or exercising woman when evaluating the risk for exercise-associated menstrual disturbances.

**Evidence-Based Statement:** The risk of developing menstrual disturbances in association with energy deficiency declines as the gynecological age increases. **Grade:** Level B.

### What Role Do Psychosocial Stressors Play in Menstrual Dysfunction?

In the context of menstrual dysfunction, psychosocial factors are often characterized as “stressors” or “challenges that individuals perceive as taxing or exceeding their coping capabilities” [168]. Such perceived stressors are believed to activate neuroendocrine pathways capable of disrupting the reproductive axis [169], although the underlying mechanisms are unclear [170]. In animal models, models of social stressors (behavioral subordination) have been shown to induce menstrual disturbances [171, 172]. Prospective studies in humans are scarce, although a significant association between perceived stress and the occurrence of anovulation and decreased progesterone was documented over two menstrual cycles in healthy premenopausal women [173]. In addition to thinking of external stressors and the perception of stressors as factors impacting reproductive function, it is important to note that chronic stress is significantly associated with increased rates of mental illness and the onset of major depressive disorder, bipolar disorder, post-traumatic stress disorders [174], and the development of anxiety sensitivity [175]. Associations between maladaptive stress-related psychiatric comorbidities and menstrual disturbances have been demonstrated by Berga et al. [176], who showed that women with functional hypothalamic amenorrhea often have psychological phenotypes suggestive of stress sensitivity and exhibit dysfunctional attitudes, such as difficulty coping with daily stressors, a high dependence on interpersonal relationships, and a high incidence of depression and anxiety [133, 166, 177, 178]. Berga et al. [176] also demonstrated that cognitive behavioral therapy could be used to successfully treat dysfunctional attitudes in women with functional hypothalamic amenorrhea and that ovarian dysfunction could be recovered in women who received such therapy [176, 179]. However, the inclusion criteria in the latter study did not include regular exercise, although some participants may have exercised.

An understanding of the extent to which psychosocial factors play a role in menstrual disturbances in athletes with, or at risk for, the Triad should take into account the extent of exposure to psychosocial “stressors” and a possible link to menstrual disturbances, as well as the association between stress-related mental health morbidities and menstrual disturbances. Regarding exposure to stressors, a competitive sports season is a documented source of physical, emotional, psychosocial, and sports-specific stressors that can have negative effects on athletes’ overall health, well-being, and performance [180–182]. That said, studies that document *direct* associations between stress exposure and menstrual disturbances in energy-replete athletes are lacking. Additionally, evidence that refeeding can reverse menstrual disturbances in exercising female individuals with FHOA [25, 38] suggests that an independent contribution of psychosocial stress is not a key factor in reproductive suppression because recovery was accomplished by reversing energy deficiency alone versus providing an intervention such as behavioral therapy designed to ameliorate psychosocial stress effects. To definitively address whether psychosocial and sport-specific stressors *independently* contribute to FHOA in exercising women, RCTs that experimentally manipulate chronic psychosocial/sport-specific stress exposure independent of energy status in exercising women are necessary. Such studies in humans would understandably be very difficult. Alternatively, in a non-human primate model of exercise-associated FHOA, the effects of psychosocial stress alone and in combination with exercise/diet were tested [183]. Conducted over two control followed by two experimental menstrual cycles, this study included one group of non-exercising monkeys exposed to a known psychosocial stress of being moved to a novel housing environment, one group that was exposed to moderate exercise combined with moderate caloric restriction, and one group that was exposed to both the psychosocial stress paradigm and exercise combined with caloric restriction. Psychosocial stress alone, and exercise combined with caloric restriction had a minimal disruptive impact on menstrual function as measured by changes in the cycle, and follicular and luteal phase lengths, but the combination of psychosocial stress and exercise combined with caloric restriction, significantly lengthened the cycle and follicular phase lengths compared with either stressor alone [183]. While this study does not isolate an *independent* effect of psychosocial stress on reproductive outcomes in an exercise model, it does experimentally demonstrate that seemingly mild/moderate stressors can combine to suppress reproductive function [183].

Stress may also act *indirectly* to impact reproductive function through its association with mental health issues and associated ED/DE and resulting energy deficiency. Although not demonstrated in athletes per se, stress has been identified as a central factor in the development, exacerbation, and maintenance of EDs [184–187]. In individuals with an ED compared to healthy controls, dysregulations of both stress axes have been documented by altered heart rate variability and salivary alpha-amylase, indicating altered SNS activation [188, 189], and altered secretion of corticotrophin-releasing hormone, adrenocorticotropic hormone, and cortisol pointing to altered hypothalamic–pituitary–adrenal axis functioning as a feature accompanying active EDs [190, 191]. While the latter studies were largely involving patients with EDs in a clinical setting, it is likely that EDs in athletes are associated with similar stress-related endocrine profiles. Moreover, given that college-aged endurance and aesthetic athletes are at an especially increased risk for ED/DE (24% and 42% prevalence, respectively [88, 192]) and the nature of ED/DE in athletes is more likely to be characterized as restrictive given its high prevalence in leanness, aesthetic, and weight class sports [96, 97, 193], it is highly plausible to link stress to menstrual disturbances, albeit indirectly, via its association with ED/DE and accompanying energy deficiency. Indeed, the stressful competitive season is often associated with the rise of EDs [194–197], concomitant with the occurrence of severe energy deficiency [3, 198]. As such, we conservatively position psychosocial stress with respect to the Triad model as an important modifying factor to consider in association with the development of ED/DE, ED/DE-associated energy deficiency, and energy deficiency-associated menstrual disturbances, as opposed to positioning psychosocial stress as a significant cause of menstrual disturbances *in the absence* of energy deficiency.

It should be noted that research in this area is challenging because “stress” and “stressors” are difficult to define and quantify, and, as noted by Loucks and Redman, stress and energy deficiency co-exist in many experimental models of stress [199]. In female athletes, many factors associated with psychosocial stress likely co-exist alongside energetic challenges associated with training, making it difficult to tease apart the drivers of reproductive disruption. Experimental interventions involving psychosocial stressors are often accompanied by energy deficiency due to stress-induced declines in appetite and food intake or the fact that some experimental stress manipulations limit access to food, for example, physical restraint stress imposed by keeping rodents in a tube [200]. As well, the fact that energy deficiency also activates the hypothalamic–pituitary–adrenal axis [68, 201, 202] underscores the challenges of teasing out independent effects. In many animal studies of psychosocial stress where an actual stress intervention can be imposed, food intake, body weight, or other metabolic changes are not quantified or reported [199]. Prospective studies quantifying both stress and energy deficiency in exercising women with a focus on menstrual function are lacking, and there are no studies where a controlled chronic stressor has been employed. However, in a prospective study of young exercising women exposed to caloric restriction, psychosocial stress was assessed using the Perceived Stress Scale [203] and 24-h repeated blood sampling was employed to capture the intervention-induced suppression of LH pulse frequency and an increase in cumulative cortisol levels assessed by area under the curve [201]. No relationship was observed between changes in psychosocial stress and declines in LH pulse frequency, but the decline in LH pulse frequency was significantly associated with an increase in cortisol area under the curve [201] likely attributable to energy deficiency-induced weight loss. While this study did not demonstrate an influence of perceived stress on the induction of perturbations in pulsatile gonadotropin profiles in response to exercise combined with caloric restriction, the participants were untrained women, not athletes presenting with menstrual dysfunction, and only one measure of stress was employed. The challenge of teasing out independent and or combined effects of psychogenic versus metabolic factors associated with menstrual disturbances in exercising women has been recognized [204, 205], emphasizing the need for prospective studies that more comprehensively quantify stress exposure *and* energetic factors to test whether an independent role for psychosocial stress exists in the induction and recovery of Triad-related reproductive dysfunction.

**Evidence-Based Statement:** Psychosocial factors should be considered as influencing the development of energy deficiency-associated menstrual disturbances. **Grade:** Level B.

### What is the Definition of Reproductive Recovery?

The concept of “reproductive recovery” is not consistently defined in the literature and varied definitions have been utilized to define the concept of “reproductive recovery” [156], including (1) at least one menses or menstrual cycle [206, 207], (2) two [157, 206, 208–210] or three consecutive menstrual cycles [211], (3) menstrual cycles of < 36 days for at least 3 months [212], and (4) three or more menses in 6 months [213, 214]. Reproductive recovery has also been defined by using thresholds of ovarian steroid concentrations observed during the recovery time period that is monitored [176]. In many cases, however, no definition of a concentration representative of ovarian steroid recovery is provided and nor is ovulation reported as having been achieved in many published studies [156]. The lack of consistent definitions for reproductive recovery creates a challenge for researchers and clinicians alike because there are no clearly defined endpoints that exercising women presenting with menstrual disturbances should be guided to achieve or to be considered “recovered.” Furthermore, simply relying on the occurrence of a single menses as an indication of recovery does not align with complete recovery of ovulation and ovarian hormone concentrations [39, 49]. Indeed, recovery of menses, when defined conservatively as two or three consecutive menstrual cycles of < 36 days, is associated with an increase in estrogen exposure [39, 49].

In the REFUEL RCT [38, 39, 49], women who improved menstrual frequency and experienced a simple recurrence of a menses event following an increased energy intake had mostly anovulatory cycles, with few menstrual cycles demonstrating ovulation and increased concentrations of ovarian hormones [39]. For example, only 31% of women with FHOA demonstrated ovulation and only 12% demonstrated evidence of progesterone activity in the luteal phase during their first recovery menstrual cycle of < 36 days [39]. It was not until women experienced three consecutive menstrual cycles < 36 days in length that a significant increase in estrogen exposure was observed from baseline to the recovery menstrual cycle [39, 49]. In direct support of our findings are data collected in other women with FHOA after a similar 6-month diet intervention of 360 kcal/day, which resulted in the resumption of menses and ovulation in 7/8 exercising women with menstrual disturbances [215]. Additionally, in support of our conclusions, Dueck et al. [40] and Kopp-Woodroffe et al. [41] state that the resumption of menses following FHOA requires a “mature” definition of resumption of at least several regular menstrual cycles of 36 days or less occurring in a period of 3 months or more. Other data in support of these findings are data collected in (1) anorexic women who recovered menstrual function within an average of 18.1 months [43,216], and (2) anorexic girls, aged 12–18 years, 9 months after menstrual recovery and weight gain who demonstrated an improvement in lumbar spine BMD [44] and, in both cases, improvements to lumbar spine BMD were proposed to be likely secondary to increased estrogen exposure-associated menstrual recovery [44, 216], and (3) in anorexic women, 20 weeks after recovering, who demonstrated resumed menstrual cycles and significantly increased estradiol over 200% [42]. Therefore, multiple consecutive menstrual cycles are likely necessary to realize improvements in the estrogen status, and the simple occurrence of menses or an increase in menstrual frequency, while an important step in the recovery journey, does not indicate adequate reproductive recovery [38–42, 49, 216].

It is also important to consider that a relapse, or a recurrence of menstrual irregularities can occur, delaying reproductive recovery and necessitating longer periods of observation with the goal of sustained menstrual regularity. We also caution against the use of identical targets to assess progress to recovery in amenorrheic versus oligomenorrheic exercising women, as the process and timeline of achieving complete recovery may be different, given that oligomenorrheic women are experiencing some menses at inconsistent and irregular intervals, and that some oligomenorrheic women may have elevated FAI [47]. As such, reproductive recovery in oligomenorrheic women with evidence of hyperandrogenemia may present differently from that observed in women with FHOA with classic suppression of hypothalamic origin.

**Evidence-Based Statement:** The simple resumption of menses or an increase in menstrual frequency, while an important step in the recovery journey, does not always indicate complete reproductive recovery. **Grade:** Level A.

**Evidence-Based Statement:** A healthy target for an exercising woman recovering from FHOA is the onset of menses followed by multiple consecutive cycles (we recommend greater than two cycles of < 36 days). **Grade:** Level A.

**Evidence-Based Statement:** Increases in estrogen exposure in exercising women recovering from FHOA are unlikely until three consecutive cycles of < 36 days are achieved. **Grade:** Level A.

### What is the Role of Energy Intake in Reproductive Recovery?

The reversal of amenorrhea or oligomenorrhea, when defined simply as the resumption of menses (amenorrheic women) or an increase in menstrual frequency (oligomenorrheic women), may occur relatively quickly after a modest increase in energy intake. In exercising women with FHOA participating in the REFUEL RCT, a modest increase in daily energy intake (330 ± 65 kcal/day, + 18 ± 4%) for 12 months was sufficient to improve menstrual regularity, which occurred in concert with increased body weight (4.9%), fat mass (18%), and TT_3_ (16%) [38]. In this RCT, women with FHOA who increased their energy intake were almost twice as likely to experience menses compared with women with FHOA in the control group who did not increase their energy intake [38]. Increases in body weight, BMI, fat mass, and percent body fat were positively associated with achieving reproductive recovery [38]. These results support the findings of several case studies [41, 206, 217] and a retrospective study [213], which previously demonstrated that an increase in body weight is associated with improved menstrual frequency in female athletes with FHOA. A 6-month prospective nutritional intervention that increased energy intake by 360 kcal/day contributed to an average body weight increase of 1.6 kg, and the reproductive recovery that occurred did so an average of 2.6 months after the start of the nutritional intervention [215, 218]. During a 9-month intervention of increased energy intake, reproductive recovery occurred in 24% of exercising women, which coincided with an increase in EA despite no significant change in body weight [219]. Other indicators of energetic status, such as increased serum IGF-1 and leptin, are also associated with reproductive recovery [42, 220]. Based on these findings, an increase in energy intake between 300 and 600 kcal/day is adequate to initiate reproductive recovery in FHOA exercising women [38, 215, 219], which aligns with the recommendation in our prior Consensus Statement [1–3]. Notably, the time course to recovery may be longer in women with a greater duration of menstrual dysfunction [215]. For example, amenorrheic exercising women undergoing a nutritional intervention required, on average, 2.63 months (with a range of 1–7 months) for the onset of menses; those reporting amenorrhea for < 1 year (*n* = 5) versus > 1 year (*n* = 2) required 1–2 months versus 6 months, respectively, for menses to resume [215]. In the REFUEL RCT, exercising women with FHOA undergoing a 12-month refeeding intervention required, on average, 6 months (range of 2–11 months) to demonstrate recovery of two consecutive eumenorrheic menstrual cycles of < 36 days [39].

Examination of menstrual frequency relative to ovarian hormones and ovulation has revealed the following: (1) simple resumption of menses (single occurrence of menses or one cycle < 36 days) did not consistently translate to improved estrogen or progesterone exposure or the presence of ovulation; (2) menstrual frequency denoted by two consecutive cycles of < 36 days was also inadequate to demonstrate a significant increase in ovarian hormone exposure; and (3) estrogen exposure significantly increased only among those achieving three or more consecutive cycles of < 36 days, indicating that multiple cycles may be necessary for improved ovarian hormone exposure [39, 219]. Moreover, the proportion of ovulatory recovery cycles observed increased with more than one consecutive cycle of < 36 days; however, a large percentage of recovery cycles in the REFUEL study were not ovulatory [39], and may require either a higher energy intake than that utilized in the REFUEL study (average 330 kcal increase per day) or a longer duration than 12 months for consistent recovery of ovarian steroids and ovulation, or a combination of both.

Although increases in body weight and fat mass have been associated with improvements in menstrual regularity [206, 212, 217, 218], the likelihood of reproductive recovery may also be influenced by baseline body composition prior to initiation of dietary intervention. In the REFUEL RCT, higher baseline fat mass was associated with a greater likelihood that the woman would experience menses during the intervention [38].

**Evidence-Based Statement:** An increase in energy intake between 300 and 600 kcal/day is often adequate to initiate reproductive recovery (i.e., increased frequency of menses) in exercising women with FHOA*. ***Grade:** Level A.

**Evidence-Based Statement:** The time course to reproductive recovery may be longer in women with a greater duration of menstrual dysfunction. **Grade:** Level A.

**Evidence-Based Statement:** Reproductive recovery with increased energy intake is more likely in exercising women with FHOA with higher baseline fat mass*. ***Grade:** Level A.

### What are the Practical Issues Affecting Menstrual Function and Recovery?

An informative qualitative study that provided important insights into athletes’ perceptions of amenorrhea reported common themes, such as (1) a failure to perceive the absence of menses as abnormal; (2) the attitude that amenorrhea is convenient; (3) prioritization of performance and the opinion that menstruation is problematic; (4) denial of amenorrhea as a basis for the medical team to prescribe alterations in diet and or training; (5) the fear that coaches and trainers are not educated adequately on the consequences of chronic amenorrhea, and the perception that discussion of menstrual problems is “taboo;” (6) the perception that not all doctors are appropriately informed on the proper medical management of menstrual dysfunction; and (7) the need for more education of athletes via various means, including social media, requiring education courses for coaches and trainers, and highlighting a multi-disciplinary treatment team [221–226]. It appears that despite all efforts towards education to date, there is still much more work necessary to improve the education of athletes, coaches, trainers, and others on the healthcare team about menstrual cycle problems in athletes, their consequences, and strategies to remedy such problems. Recent efforts on social media platforms targeting key stakeholders hold promise for improved awareness and education [227].

**Evidence-Based Statement:** Many athletes’ perceptions of amenorrhea and/or exercise-associated menstrual disturbances reflect a poor understanding and/or a denial of the severe health consequences of amenorrhea. **Grade:** Level C.

## Scientific Updates to the Bone Health Continuum

### General Comments

Both energy deficiency and suppression of the HPO axis have been well documented to negatively impact bone health in exercising adolescent girls and young adult women with FHOA. This manifests as alterations in bone turnover [228–231] and in clinically relevant endpoints, such as low areal [53, 54, 232] and volumetric BMD and altered bone geometry [53, 56, 233–235]. Below, we summarize updated evidence for the effects of the Triad on metabolic bone turnover, structural parameters such as density, geometry, microarchitecture, and strength, and musculoskeletal injury outcomes, including BSI.

### What is Currently Known About Bone Outcomes Secondary to Energy Deficiency With or Without the Presence of FHOA?

#### Evidence on BMD, Geometry, Microarchitecture, and Strength

Athletes with the Triad often present with low BMD, defined as a BMD Z-score < − 1.0 for weight-bearing sports [18]. This is an even greater concern in athletes with a family history of osteoporosis [236]. In adolescent and young adult athletes with FHOA, lower areal BMD has been reported at the spine, hip, and total body, with the greatest differences observed at the lumbar spine, a site rich in estrogen-sensitive trabecular bone [53–55, 237, 238]. With respect to bone geometry and structure, differences in both the trabecular and cortical compartments have been observed in exercising adolescent girls and young adult women with FHOA [56]. At the distal radius and distal tibia, athletes with FHOA have higher cortical porosity and lower cortical thickness and cortical volumetric BMD than their non-athlete counterparts, and lower strength estimates (stiffness and failure load) consistent with an increased fracture risk compared with both non-athletes and eumenorrheic athletes [56]. Cortical porosity is higher, and trabecular volumetric BMD and strength estimates at the tibia are lower in athletes with a history of two or more stress fractures than those with fewer than two stress fractures [56]. Trabecular morphology and alignment are also impaired in athletes with FHOA with a history of recurrent stress fractures, including lower plate-like trabecular bone volume fraction, fewer axially aligned trabeculae, decreased plate number and plate thickness, and less trabecular connectivity at the distal radius [55]. Importantly, in an observational study over a 12-month time period, athletes with FHOA demonstrated lower hip BMD Z-scores, lower radial and tibial failure loads, and failed to “catch-up” to eumenorrheic athletes and healthy non-athletic controls, despite the mechanical loading imposed on bone in these exercising women, and despite the resumption of menses in about 40% of these young women [57]. Moreover, during acute severe body mass loss in highly active women, loss of bone from the axial skeleton is observed, while tibial bone is protected [239]. These findings indicate a cause for concern in that the athletes with FHOA may not consistently benefit from the typical adaptive forces of weight-bearing exercise and mechanical loading on bone, likely because of hypoestrogenism and energy deficiency, resulting in an increased risk of fracture [56, 57].

Studies have further aimed to differentiate the impact of energy versus estrogen deficiency on BMD, bone geometry, and estimated bone strength, and demonstrated that energy-deficient versus energy-replete women had lower distal tibial total and trabecular volumetric BMD, cortical area, and estimated compression strength, as well as lower proximal tibial total volumetric BMD and cortical area and thickness, and a larger endosteal circumference [234]. Compared with normoestrogenic women, estrogen-deficient women had lower distal radius total and cortical volumetric BMD, a larger total and trabecular area, lower estimated compression strength, and lower proximal radius cortical volumetric BMD [234]. These data suggest greater effects of energy deficiency at weight-bearing sites, and greater effects of estrogen deficiency at non-weight-bearing sites. Lower BMD and impairment in other bone endpoints in adolescent and young adult athletes have also been associated with lower BMI [52, 55, 240], lean mass [52, 53], android/gynoid body fat ratio [241], IGF-1 [52], leptin [242], and irisin levels [243], older menarcheal age [53–55], a longer duration of amenorrhea [52], and greater visceral/subcutaneous fat ratio [237], spine marrow fat [237], drive for thinness [244], EDI score [138, 244], cognitive eating restraint [244], intake of dietary fiber [245], and peptide YY levels [242].

**Evidence-Based Statement:** Athletes with FHOA demonstrate lower BMD, and impaired bone geometry, structure, and strength estimates compared with athletes with eumenorrhea and non-athletes, particularly at non-weight-bearing sites; energy deficiency has a greater impact on these parameters at weight-bearing sites. **Grade:** Level A.

#### Evidence on Bone Turnover

The relationship between estrogen deficiency, energy deficiency, and bone turnover in women is well established [228–231, 246]. Energy deficiency is associated with suppressed bone formation, as has been demonstrated in cross-sectional studies of energy-deficient exercising women quantified by measures of suppressed RMR, low TT_3_ concentration, and often overtly indicated by menstrual disturbances (FHOA) [228, 229, 231, 246]. Similar associations have been demonstrated in prospective experimental studies. In a laboratory setting of controlled EA, reducing EA to 30 kcal/kg lean body mass (LBM)/day or less resulted in suppression of bone formation markers in a dose-dependent manner [230]. Similarly, dietary restriction resulting in EA of 15 kcal/kg FFM/day for 3 days reduced bone formation, while EA of 15 kcal/kg FFM/day resulting from increased exercise energy expenditure did not alter bone turnover [247]. Markers of bone resorption demonstrate energy-related responses as well, with cross-sectional reports in exercising women demonstrating either increased or decreased [228, 229] concentrations of bone resorption markers. Experimental manipulation of energy to achieve EA of 10 kcal/kg LBM/day resulted in a significant increase in bone resorption [230]. Indicators of energetic status, such as TT_3_ and RMR, were positively associated with bone resorption markers in exercising women [228]. Estrogen/menstrual status, which is also directly impacted by energy deficiency, may further contribute to alterations in bone turnover associated with the Triad, considering the known inhibitory relationship between estrogen and bone resorption. In a cross-sectional study of estrogen-deficient (amenorrheic and some oligomenorrheic) and estrogen-replete (eumenorrheic) exercising women, women who were both estrogen and energy deficient demonstrated significantly greater bone resorption than estrogen-replete women both with and without energy deficiency [231]. In the same study, estrogen status explained 15% of the variance and RMR explained 20% of the variance in bone resorption, highlighting the combined roles of energetic and estrogenic status on bone resorption [231].

Overall, the perturbations in bone formation and resorption that occur with energy deficiency, especially when energy deficiency leads to estrogen deficiency and menstrual disturbances, likely contribute to the structural detriments in bone evident in women with the Triad (e.g., reduced BMD). Of note, even acute periods of energy deficiency have been associated with perturbations in bone turnover markers, similar to those reported with chronic energy deprivation, highlighting the significant impact of energy status on bone turnover.

**Evidence-Based Statement:** Athletes with FHOA demonstrate alterations in bone turnover markers consistent with their estrogen and/or energy status. **Grade:** Level A.

### What is Known About BSI in Exercising Women and Athletes with Energy Deficiency and Menstrual Disturbances?

Bone stress injuries in trabecular-rich bone, such as the pelvis, femoral neck, or calcaneus, exhibit larger associations with factors consistent with energy deficiency. Data from a chart review of 127 female athletes, age 15–30 years, presenting for BSI diagnosis demonstrated that athletes with BSIs at a site higher in trabecular-rich bone were more likely to meet criteria for energy deficiency (as determined from surveys evaluating ED/DE risk) and exhibited more Triad risk factors than athletes with BSIs at cortical-rich sites [248]. In addition, female athletes with FHOA had BSIs of higher magnetic resonance imaging (MRI) grades compared with eumenorrheic athletes [249] and a lower BMD may be more strongly associated with BSI in trabecular-rich locations, resulting in a longer time for return to sport [249, 250]. However, a recent study evaluating the trabecular bone score did not enhance prediction for elevated BSI risk among female collegiate athletes [251]. Trabecular bone score is measured from dual-energy x-ray absorptiometry images using variations in grayscale and texture believed to serve as a surrogate for bone microarchitecture, a determinant of bone strength independent of BMD. Unlike high-resolution peripheral quantitative computed tomography, trabecular bone score may not accurately reflect bone microarchitecture.

Based on the evidence that has accumulated surrounding BSIs in relation to energy deficiency and menstrual disturbances [32, 56, 233, 252], the Triad model has been expanded to include BSI on the bone health spectrum. Specifically, low-risk BSI is considered a subclinical indicator of the Triad and high-risk or recurrent BSI is considered a pathological indicator. High-risk sites are defined as the femoral neck, sacrum-pelvis, calcaneus, tarsal navicular, anterior tibial shaft, and fifth metatarsal.

**Evidence-Based Statement:** Evidence of energy deficiency and FHOA are important determinants of BSIs in athletes. **Grade:** Level A.

### What are the Triad Risk Factors for Poor Bone Health in Athletes and Exercising Women?

Risk factors for impaired bone mass in female athletes include underweight status [52, 55, 240], lower lean mass [52, 53], lower android/gynoid body fat ratio [241], higher visceral/subcutaneous fat ratio [237, 244], ED/DE, elevated drive for thinness and cognitive eating restraint [244], participation in a leanness sport [253], drinking less milk, greater intake of dietary fiber [245], later age of menarche [53–55], longer duration of amenorrhea [52], menstrual irregularity [53–55, 244], prior history of fracture, and alterations in hormones (lower IGF-1, leptin and irisin, higher PYY) [138, 241, 253, 254]. In an evaluation of 320 female adolescent athletes, several indicators of underweight status were associated with low BMD, including BMI < 18.5 kg/m^2^, BMI less than the 5th percentile-for-age (Centers for Disease Control and Prevention pediatric growth chart), and < 85% of expected weight [254]. A body fat percentage Z-score < − 1.0 was also associated with a BMD Z-score < − 2.0 [254]. Specifically, among adolescent runners, girls with a lower android/gynoid ratio, later age of menarche, lower lean mass, drinking less milk, or both menstrual irregularity and a history of fracture were significantly more likely to exhibit low bone mass [241]. In a study of exercising girls and women, accumulation of Triad risk factors, including low BMI (< 18.5 kg/m^2^), late age at menarche, current FHOA, elevated dietary restraint, and participation in a leanness sport, was associated with an increased risk of low BMD [253]. Knowledge of these risk factors, including an assessment of the family history of osteoporosis [255], can guide sports medicine professionals in evaluating and managing female athletes at risk for impaired bone health, especially as a large genetic component of BMD exists, with heritability of BMD suggested to be as much as 50–85% (site dependent) [255].

All athletes exhibiting Triad risk factors are at elevated risk for lower BMD, regardless of sport, but specific attention may be required for adult athletes with low BMI (BMI < 18.5 kg/m^2^) or current FHOA, as evidence suggests that these risk factors are the strongest predictors for low BMD [241]. Although BMI may not be a perfect measure of body composition and energy status, it remained a more robust predictor for low BMD (Z-score < − 1.0) compared with lean mass or fat mass [241, 254]. A BMI is easier to quantify than body composition, and measures of both menstrual function in the past year and a BMI may be important to screen for in female athletes to determine who is at higher risk for low BMD.

**Evidence-Based Statement:** The risk of low BMD is higher in exercising girls and women as the number of Triad risk factors increases (cumulative risk). **Grade:** Level A.

### What are the Associations Between Triad Risk Factors and BSIs?

Prior research indicates notable associations between Triad Cumulative Risk Assessment (CRA) scores and BSIs. A study evaluating an elite collegiate sports program demonstrated that 29% of female athletes were classified into a moderate- or high-risk category on the CRA and had a 2.6- and 3.8-fold increased risk for BSIs, respectively, compared with low-risk category athletes [256]*. *A study of national and Olympic middle- and long-distance runners and race walkers also reported a significantly higher number of lifetime fractures in athletes meeting CRA criteria for moderate and high risk compared with low risk [257]. Another study of collegiate distance runners found that the presence of Triad risk factors in an athlete might result in more significant time to recover from BSI [249].

**Evidence-Based Statement:** The risk of a BSI is higher in exercising girls and women as the number of Triad risk factors increases (cumulative risk). **Grade:** Level A.

In a multi-site prospective study of collegiate runners, female runners considered high risk based on Triad CRA scores had a four times greater rate of trabecular-rich BSIs compared with runners with low-risk scores. Of the individual CRA score components, low BMD and FHOA risk scores were most predictive of trabecular-rich BSIs [258]. In a cohort study of 321 female elite athletes, the Triad CRA score was significantly associated with both trabecular- and cortical-rich BSIs [259]. In this study, lower BMD and lower weight were associated with an increased risk for experiencing trabecular-rich BSIs compared with no BSI (odds ratio per standard deviation lower = 3.82 for spine BMD; 1.84 for total body BMD; 4.55 for weight) and a greater risk for trabecular-rich BSIs compared with cortical-rich BSI (odds ratio = 3.08, 2.38, and 5.26, respectively), whereas being taller was associated with an increased risk for cortical-rich BSI compared with no BSI (odds ratio per standard deviation higher = 2.14) and an increased risk for cortical-rich BSI compared with trabecular-rich BSI (odds ratio = 3.57) [259].

**Evidence-Based Statement:** Female athletes with a higher cumulative Triad risk have a greater risk for trabecular-rich BSIs compared with cortical-rich BSIs. **Grade:** Level B.

Although some studies in young women who sustained a BSI identified lower BMD as a risk factor [32, 56, 258–260], particularly low BMD of the spine and total body [56], not all athletes who sustain a BSI have low BMD, suggesting that risk factors for BSI are complex and likely multifactorial. For example, bone geometry and structure are likely to play a major role. In one study, amenorrheic athletes with two or more stress fractures also had lower total cross-sectional area, trabecular volumetric BMD, stiffness, and failure load at the radius, and lower stiffness and failure load at the tibia compared with those with fewer than two stress fractures [56].

## Introducing an Adolescent Triad Model

### General Comments

Adolescence, the transition from childhood to adulthood from age 10–19 years, is a key time period of maturation and development that sets the foundations for good health across the lifespan [137]. Adolescent athletes require adequate nourishment to support normal growth, pubertal development, and bone mineralization, and therefore they are particularly vulnerable to the impacts of energy deficiency [261]. Female adolescent athletes may be impacted by the Triad, which underscores the need for early identification, treatment, and prevention efforts.

The Female Adolescent Triad Model presents a framework of three spectra of the Triad in adolescent athletes. The model includes the optimal health, subclinical, and pathological criteria, adjusted to align with the physiology and development of adolescent athletes, for each of the three Triad components (Fig. 4a). Further detailed criteria for the healthy, subclinical, and pathological categories for each component are provided in Fig. 4b. Prior to the publication of the 2025 Triad Consensus Statement, researchers and practitioners working with female adolescent athletes translated recommendations and guidelines from the existing adult Triad model [1–3]. This posed challenges owing to the specific nature of adolescent development, which includes the onset of menses and the rapid bone mineralization occurring early in the second decade of life [262]. While, on average, menarche begins in girls at age 12.4 years [263], it can take up to 2 years for the menstrual cycle to normalize. Therefore, some menstrual cycle irregularities can be normal during the initial post-menarcheal stage, which is addressed in the adolescent model. While the adult model provides the criteria for determining underweight status in adults, the Female Adolescent Triad Model describes BMI as a percentile-for-age using the Centers for Disease Control and Prevention pediatric growth chart [263]. The International Society of Clinical Densitometry definition for osteoporosis also differs between pediatric and adult populations [264]. Additionally, the seminal research studies providing evidence of the dose–response relationship between EA and biomarkers of bone turnover, reproductive, growth, and metabolic hormones were conducted in young adult women [24, 155, 230]. Preliminary evidence suggests that the endocrine system of adolescents may be more sensitive to energy deficits [167]. Guidelines that consider growth trajectory, onset of menarche, normalization of menses, and bone mineral accrual are recommended while assessing and treating female adolescent athletes. This is an initial working model, based on the current evidence. Future research is needed to further clarify these critical areas impacting the treatment and management of the Triad in adolescent athletes.

### What Growth and Developmental Changes Occur During Adolescence?

The adolescent years, which include puberty, represent a unique period of growth and development. In girls, puberty is characterized by the pubertal growth spurt, development of secondary sexual characteristics, onset of menarche, and an increased rate of bone accretion and mineralization [265]. Peak height velocity and weight gain precede menarche, with a linear growth rate averaging ~ 8.3 cm and weight gain of approximately 3 kg per year [266]. A rapid increase in bone mineralization occurs after peaking linear growth. In girls without delayed onset of menarche, this corresponds to an average age range for peak bone mineral content (BMC) accumulation between ages 11.5 and 13.5 years [267]. Bone mineralization continues, though at a slower rate, throughout the second decade of life, with 40–50% of adult BMC accrued between ages 11 and 18 years, and more than 80% of peak bone mass accrued by the end of the second decade [267–269]. It is thus essential to ensure that conditions are optimized for bone accrual during these critical pre-teen and teenage years.

**Evidence-Based Statement:** The adolescent years are a critical time of life to optimize bone accrual and overall health. **Grade:** Level A.

### How Long Does it Take to Achieve Menstrual Maturity in Adolescents?

In the first few years after menarche, it is not unusual to observe menstrual cycle irregularity and anovulation [270, 271], so it can be difficult to determine the presence of menstrual disturbances due to energy deficiency. Indeed, it often takes 2–3 years to achieve regular cyclicity and it is not until the fourth year after menarche (year 4 of gynecological maturity) that the prevalence of irregular menstrual cycles drops below 10% [272–274]. About 50% of the cycles observed during the first 2 years after menarche are anovulatory, and it is not until year 5 post-menarche that about 75% of the cycles are ovulatory and menstrual cycle length in 95% of these adolescents is 21–40 days [272–274]. Normal menstrual cycle length (< 36 days) is reportedly not established until approximately the sixth gynecologic year, which is equivalent to a chronologic age of approximately 19 or 20 years in girls without delayed menarche [275]. Moreover, the consistency of ovulation from one menstrual cycle to the next is dependent upon the time since menarche and the age at menarche, such that menarche at an early age is associated with early onset of ovulatory cycles, whereas later onset of menarche is associated with delayed establishment of ovulatory cycles [276, 277]. According to the American Academy of Obstetrics and Gynecology, delayed menarche is defined as no menses by age 14, primary amenorrhea as no menses by age 15 years or thereafter, and secondary amenorrhea, the absence of menses for more than 90 days after the establishment of regular menstrual cycles [274, 278].

**Evidence-Based Statement:** We concur with defining primary amenorrhea as no menses by age 15 years, and secondary amenorrhea as the absence of menses for more than 90 days after 1–2 years post-menarche. **Grade:** Level A.

### What is the Evidence for an Effect of Energy Deficiency and Nutrient Deficits During Adolescence on Pubertal Growth and Menstrual Cycles?

Adequate intake of energy, protein, essential fatty acids, vitamins, and minerals plays a key role in supporting linear growth, pubertal development, and bone mineralization. Female adolescent athletes expend higher levels of energy, compared with non-athlete peers, because of regular exercise training and, thus, require a higher energy intake, which ranges from 2300 to > 3000 kcal/day [279]. Although adequate nutrition and expected weight gain during childhood support pubertal development, underfeeding has been associated with delayed onset of puberty. It is well established that the process of puberty and reproductive maturation and growth, to include attainment of peak height, peak weight, and peak bone mass, is highly dependent on nutrition and adequate energy intake [280–283]. A diet that is deficient in calcium and vitamin D could also adversely impact bone mineralization [283]. Further, a higher intake of dietary fiber and vegetable protein has been associated with lower bone density in athletes [245].

Chronic undernutrition may delay menarche. Athletes who engage in leanness sports and aesthetic athletes (e.g., gymnasts, figure skaters) are more likely to have delayed menarche, and both primary and secondary amenorrhea [284]. Menstrual disturbances are also common in post-pubertal female athletes with an older gynecological age in a state of energy deficiency [280–283], and the Triad is often observed in exercising girls with energy deficiency (with or without DE).

Energy deficiency may affect linear growth in adolescents. Whether height is impacted depends on the period during which the athlete is exposed to energy deficiency. If energy deficiency occurs early in life before pubertal onset and before the occurrence of the pubertal growth spurt, height could be affected deleteriously [285]. However, a delay in menarche may be observed without concomitant effects on attainment of adult height [286, 287]. Energy deficiency also may affect bone mineral accrual. Adolescents with a clinical ED [240], elevated dietary restraint [244], those engaged in leanness sports (such as running) (207) [253], and those with underweight status [52, 55, 240] and associated lower lean mass [52, 53], higher spine marrow fat [237], later menarcheal age [53, 54], early menarcheal age [288], longer duration of amenorrhea [52], and lower IGF-1 [52], leptin [242], irisin [243] and higher PYY concentrations [242], exhibit low total body and lumbar spine BMD and BMC, impaired bone geometry and microarchitecture, lower bone strength estimates, reduced pubertal bone accrual, and are at risk of attaining low peak bone mass [289].

**Evidence-Based Statement:** Energy deficiency and suboptimal nutrient intake in adolescent female athletes can result in delayed menarche, primary or secondary amenorrhea, low BMD, reduced bone accrual, and low peak bone mass. **Grade:** Level A.

### What is the Prevalence of the Triad in Female Adolescent Athletes?

To date, two studies have evaluated each component of the Triad in female adolescent athletes representing a variety of sports [290, 291]. Estimates of DE or EA < 30 kcal/kg FFM/day in these investigations ranged from 4 to 18%, menstrual irregularity (FHOA) from 24 to 54%, and low BMD (*Z*-score < − 1.0) from 16 to 22% [290, 291]. Another study evaluated each component of the Triad in 78 female adolescent swimmers and reported DE, menstrual irregularity, and low BMD in 45%, 19%, and 15% of athletes, respectively [292]. Another investigation of 311 adolescent athletes participating in a variety of sports reported a 35% prevalence of DE and a 19% prevalence of menstrual irregularity [293]. These studies suggest that a significant proportion of adolescent athletes may exhibit one or more components of the Triad.

Additional studies report estimates of one or two Triad components, further addressing the impact of the Triad on female adolescent athletes. Studies specifically evaluating EA vary in the proportion of athletes exhibiting an EA level < 30 kcal/kg FFM/day, with estimates that range between 6 and 58% [290, 294–296]. A study evaluating EA in 185 adolescent female athletes across a range of sports, mean age 16.3 ± 2.8 years, reported that 51% of athletes had EA < 30 kcal/kg FFM/day [290, 297]. Additional investigations report EA < 30 kcal/kg FFM/day in adolescent soccer players (53–58%) [294, 298], endurance runners (18–28%) [296, 299], rhythmic gymnasts (45%) [295], and ballet dancers (22%) [300]. The range in estimates of EA < 30 kcal/kg FFM/day may be due to the variety in methods used to evaluate each factor (i.e., energy intake, exercise energy expenditure, FFM) and sport types represented, as leanness sports exhibit a higher risk for low EA and subsequent outcomes [78]. It should be noted that the abovementioned studies utilized measurements of EA as an indicator of energy status. Going forward, research on the prevalence of energy deficiency in adolescent athletes should include additional measurements of energy status because of concerns regarding the variability and difficulty in accuracy of EA assessments.

Several studies describe higher estimates of DE in adolescent athletes participating in aesthetic, weight-class, or endurance sports compared with ball game sports [301, 302]. Other investigations report no association between DE and sport type [293, 303]. While primary amenorrhea (i.e., age at menarche ≥ 15 years) is observed in approximately 2% of the general population [304], higher estimates have been reported for adolescent athletes in competitive soccer [256, 305, 306], endurance running [241, 256, 296, 306], swimming (~ 20%) [256, 306], and rhythmic gymnastics (up to 54%) [256, 284, 306–309]. Moreover, in 423 female high school athletes, a significantly higher proportion participating in leanness, compared with non-leanness, sports met criteria for oligo- or amenorrhea [303]. Although the proportion of female adolescent athletes with low BMD (Z-score < − 1.0) ranges from ~ 16% to 22% across a variety of sports [290, 291], higher estimates of low BMD of ~ 40% are reported in female adolescent endurance runners [310]. Female adolescent athletes in repetitive or non-impact sports (middle/long-distance running and swimming) also exhibit higher estimates of low lumbar spine BMD (Z-score < − 1.0) compared with athletes participating in high/multi-directional impact sports (i.e., ball sports, track and field sprinters, field events) [311]. Adolescent ballet dancers also exhibit a risk for undernourishment, late age at menarche, and lower BMD and BMC values [312, 313].

**Evidence-Based Statement:** The prevalence of energy deficiency among adolescent female athletes is variable depending on the nature of sports activity. **Grade:** Level B.

**Evidence-Based Statement:** Adolescent athletes engaged in aesthetic, weight-class, endurance, leanness, or non-weight-bearing sports are at increased risk for late age at menarche, primary or secondary amenorrhea, and low BMD. **Grade**: Level A.

**Evidence-Based Statement:** Participation in aesthetic, weight class, endurance, and/or leanness sports may increase the risk of DE. **Grade:** Level B.

### Is the Prevalence of the Triad Higher in Adolescent Athletes Compared to Non-Athletes?

Presently, one study has compared the prevalence of the Triad between adolescent competitive athletes (*n* = 80) and non-athlete controls (*n* = 80) [290]. A higher proportion of athletes met criteria for menstrual irregularity; the prevalence of secondary amenorrhea (current or history) was two times higher in athletes (30%) versus non-athletes (15%). A higher proportion of non-athletes (30%) versus athletes (16%) exhibited BMD Z-scores < − 1.0. No differences in DE scores or subclinical or clinical low EA were observed between athletes and non-athletes [290]. Additional studies evaluating adolescent athletes compared to non-athletes also reported a lack of significant differences in DE attitudes and behaviors [314, 315]. A study evaluating more severe pathology conducted a clinical evaluation for ED/DE and identified that a higher proportion of female adolescent athletes (14%), compared with non-athlete controls (5%), met *Diagnostic and Statistical Manual of Mental Disorders* criteria for EDs [117]. In a sample of adolescent endurance athletes versus non-athlete controls, a significantly higher proportion of athletes had EA < 30 kcal/kg FFM/day (18% vs 2.2%) and menstrual irregularity (33% vs 18%); however, clinical DE scores did not differ significantly between groups [296]. Upon evaluation of the effects of sport participation on adolescent bone health, several additional studies reported higher BMD values in athletes compared with non-athlete controls [316–318]. Differences in BMD between athletes and non-athletes may also vary according to bone site and loading characteristics, with odd- and high-impact loading providing more favorable increases in bone [311].

**Evidence-Based Statement:** Adolescent athletes are at a higher risk of menstrual irregularity compared with non-athletes. **Grade:** Level B.

**Evidence-Based Statement:** In adolescents, eumenorrheic athletes or athletes participating in multi-directional impact-loading sports exhibit higher BMD values than non-athletes. **Grade**: Level A.

### How are BMD and Bone Microarchitecture Affected in Adolescents with the Triad?

Lower areal BMD has been reported at the spine, hip, and total body, with the greatest differences observed at the lumbar spine in adolescents with the Triad [53–56]. With respect to bone geometry and structure, alterations in both the trabecular and cortical compartments have been observed in exercising adolescent girls and young adult women with FHOA compared with non-athlete controls [53, 54, 56, 233, 235]. Trabecular morphology, including lower plate-like trabecular bone volume fraction, fewer axially aligned trabeculae, decreased plate number and plate thickness, and less trabecular connectivity and alignment at the distal radius, is also impaired in adolescent and young adult amenorrheic athletes with a history of recurrent stress fractures [55]. Adolescent and young adult athletes with FHOA have lower bone strength estimates at the radius compared with non-athletes, and do not demonstrate the higher bone strength estimates at the tibia compared with eumenorrheic athletes [56].

**Evidence-Based Statement:** Adolescent athletes with FHOA are at risk for low BMD, particularly at estrogen-sensitive sites, with impaired bone geometry, structure, and strength estimates. **Grade:** Level A.

### What Factors Increase the Risk of Low BMD in Adolescent Athletes?

Several investigations among female adolescent athletes address factors associated with low BMD. Factors including low BMI, DE, and menstrual disturbances are associated with an energy deficiency and support the Adolescent Triad model. Various studies have demonstrated that adolescents with a clinical ED [240] have characteristic traits, including endocrine alterations (lower IGF-1 [52], leptin [242], irisin [243], and higher PYY concentrations [242]), menstrual-related factors (later menarcheal age [53–55] and early menarcheal age [288], longer duration of amenorrhea [52]), bone health-related alterations (low total body and lumbar spine BMD and BMC, impaired bone geometry and microarchitecture, lower strength estimates, reduced pubertal bone accrual, being at risk of attaining low peak bone mass [289], and higher spine marrow fat [237]), and other characteristic findings typical of Triad-affected athletes, including elevated dietary restraint [244], underweight status [52, 55, 240], lower lean mass [52, 53], engaging in leanness sports (such as running) [253], and having a greater intake of dietary fiber and vegetable proteins [245].

In an evaluation of 320 female adolescent athletes representing a variety of sport types, several indicators of underweight status were associated with low BMD, including BMI < 18.5 kg/m^2^, BMI < 5th or < 10th percentile-for-age (Centers for Disease Control and Prevention pediatric growth chart), and < 85% of expected weight [254]. In another study of female adolescent athletes, higher BMI was associated with a reduced risk of low BMD in a multivariate model adjusting for menstrual function, EA, calcium intake, and age [289]. In an investigation evaluating 94 female adolescent runners, having a BMI ≤ 17.5 kg/m^2^, and having both a history of menstrual irregularity and a history of fracture were significant risk factors for low BMD in the multivariate model [241]. Lower BMI is a robust predictor of low BMD (Z-scores < − 1.0) [241, 254].

Research among endurance athletes provides further evidence of associations between menstrual irregularity, DE, and low BMD. In 170 female high school athletes, low BMD was associated with later age at menarche and athletes with FHOA exhibited lower trochanter BMD compared with eumenorrheic athletes [291]. Another study of 93 female adolescent runners also identified menstrual irregularity as a risk factor for low BMD (Z-score < − 1.0); higher BMI and lean tissue mass were protective against low BMD and participating in more than five endurance running seasons increased the risk [310]. Elevated dietary restraint has also been significantly associated with low lumbar spine BMD (for Z-score ≤ − 1.0 and ≤ − 2.0) in female adolescent endurance runners [319]. Furthermore, in a 3-year prospective study, adolescent runners who met criteria for DE, as assessed by the Eating Disorder Examination Questionnaire (EDE-Q), failed to gain lumbar spine or total hip BMD as compared with athletes not meeting criteria for DE. Higher EDE-Q weight concern scores were inversely associated with a lumbar spine BMD Z-score change between assessments. Runners with DE at baseline also reported significantly fewer menstrual cycles per year and more years of amenorrhea. Disordered eating, particularly dietary restraint and weight concern, may also indicate a risk of low BMD or reduced bone mineral accrual [320].

These studies emphasize the importance of adequate energetic status to promote normal body weight, and sufficient estrogen exposure, as indicated by normal menstruation during the critical period of bone mineralization, to support bone health and attainment of optimal peak bone mass.

Although all athletes exhibiting Triad risk factors are at elevated risk for lower BMD, regardless of sports, specific attention may be required for athletes with low BMI (BMI < 18.5 kg/m^2^) or FHOA, as evidence suggests that these risk factors are the strongest predictors for low BMD [241]. Body mass index as well as menstrual status in the preceding year may be important to screen for female athletes to identify those at higher risk for low BMD.

**Evidence-Based Statement:** Low BMI, and/or clinically significant weight loss, and/or tracking below the weight trajectory on the Centers for Disease Control and Prevention growth curve, may indicate energy deficiency and a risk for FHOA, both of which are key risk factors for low BMD in female adolescent athletes. **Grade**: Level A.

### Are Adolescent Athletes at a High Risk for BSIs?

According to the US National High School Sports-Related Injury Surveillance System reporting on data collected among adolescent athletes between ages 13 and 19 years at 100 randomly selected high schools between 2005–6 and 2012–13, 0.8% of injuries were characterized as a BSI, with a BSI rate of 1.54 per 100,000 athlete exposures (AEs) [321]. Female athletes exhibited a significantly higher rate of BSI compared with boys (2.22/100,000 AEs vs 1.27/100,000 AEs); sports with the highest rates reported included girls’ cross-country (10.62/100,00 AEs) and gymnastics (7.43/100,000 AEs) [321]. The rates of BSI among high school athletes (1.54/100,000 AEs) are lower than those of collegiate athletes as reported by the NCAA Injury Surveillance Program, with a BSI rate of 5.7/100,000 AEs during the academic years of 2004–5 to 2013–14 [321]. Consistent with the high school athletes, collegiate sports with the highest rates of BSI included women’s cross-country (28.59/100,000 AEs), women’s gymnastics (25.58/100,000 AEs), and women’s track (22.26/100,000 AEs) [322]. While the incidence of BSI is lower among adolescent compared with collegiate athletes, a subset of female athletes participating in cross-country, track and field, and gymnastics exhibits an elevated BSI risk.

Additionally, in a prospective study evaluating the incidence of BSI in 748 high school track and field athletes, approximately 5% of athletes reported a BSI during the 11.4-month follow-up period (2.3 total seasons of cross-country and track and field), with a higher incidence in girls compared with boys (5.4% vs 4.0%) [241]. Female sex-specific risk factors included previously sustaining a BSI, late age at menarche (≥ 15 years), BMI < 19 kg/m^2^, and prior participation in gymnastics or dance [241]. In a prospective multi-site study including 259 adolescent athletes (*n* = 151), collegiate athletes (*n* = 25), and women (*n* = 83) participating in competitive or recreational exercise activities, 10.8% sustained a BSI [32]. The subset of participants with the highest BSI incidence of 46% was participating in ≥ 12 h of purposeful exercise/week, had low BMD (< − 1.0) and three to four additional risk factors, including BMI < 21.0 kg/m^2^, oligo- or amenorrhea, elevated dietary restraint, or participation in a leanness sport [32]. A cross-sectional study of 100 athletes with FHOA, 35 eumenorrheic athletes, and 40 non-athletes reported a 32% prevalence of BSI history among athletes with FHOA compared with 6% of EAs (0% in non-athletes) [56]. A follow-up study of 468 adolescent and young adult women with anorexia nervosa (*n* = 269), FHOA (*n* = 104), and eumenorrheic controls (*n* = 95) aged 14–22 years identified a higher prevalence of prior BSI among athletes with FHOA (25%) compared with participants with anorexia nervosa (5.0%) and control participants (1.2%), despite lower total body and lumbar spine BMD Z-score values observed in participants with anorexia nervosa compared with athlete participants with FHOA [233].

Prior investigations support the multifactorial nature of BSI development among adolescent athletes with various factors (i.e., BMD, age at menarche, menstrual function, sport type, sports participation history, prior injury, eating attitudes and behaviors) moderating risk. These risk factors are consistent with observations among collegiate and young adult athletes and suggest that while the rates of BSI among adolescents are lower than collegiate athletes, traits from adolescence may influence the future BSI risk.

**Evidence-Based Statement:** Female adolescent athletes participating in cross-country, track and field, and gymnastics exhibit an elevated BSI risk affected by BMD, age at menarche, menstrual function, sport type, sports participation history, prior injury, eating attitudes and behaviors. **Grade:** Level B.

### Can Adolescent Athletes with Low BMD Normalize BMD Levels?

In a 3-year prospective study evaluating changes in bone in female adolescent runners, lumbar spine and total body BMC increased significantly from baseline to follow-up in all runners; however, 87% (13/15) of runners with low BMD (Z-score < − 1.0) at baseline continued to meet criteria for low bone mass [323]. Overall, runners with low BMD at baseline exhibited characteristics thought to favor the potential for “catch-up” bone mineralization, including an increase in approximately 5 kg of body weight and 0.4 kg in lean tissue mass. In runners with low BMD at baseline, the number of miles run between baseline and follow-up negatively related to lumbar spine BMC change [323].

Of note, two runners with low baseline BMD in the 3-year prospective study exhibited normal menstrual function between baseline and follow-up, an increase in BMI and a 1.10 standard deviation increase in the lumbar spine BMD Z-score, which changed their BMD status from “low” to “normal” [323]. This finding is consistent with two published case reports describing the 6- to 8-year recovery of adolescent runners with high levels of training, DE, and amenorrhea. The runners in the case reports exhibited a two standard deviation increase in lumbar spine BMD Z-score following a period of decreased training, weight gain, and normal menstrual function [324, 325]. These reports provide evidence for the potential significant increase in the BMD Z-score consistent with “catch-up” bone mineralization and normalization of bone mass. However, these reports are anecdotal, and it is unclear whether similar findings will be observed in larger studies. In fact, in a 12-month observational study evaluating bone mineral change among adolescents and young adults with FHOA and eumenorrheic athletes (mean age 19.2 years), athletes with FHOA demonstrated compromised bone health and failed to “catch-up” to eumenorrheic athletes and healthy non-athletic controls, despite the mechanical loading exposure from exercising and resumption of menses in about 40% of athletes with FHOA [57].

**Evidence-Based Statement:** While low BMD among female adolescent athletes appears possibly irreversible in the short term, normalization of weight and menstrual function of sufficient duration in some athletes may allow for catch-up bone mineralization and normalization of bone mass. **Grade:** Level C.

## Emerging Issues

### General Comments

In this updated 2025 Consensus Statement, we present several issues we characterize as emerging issues of concern when considering outcomes of significance related to Triad science.

### What Other Musculoskeletal Injury Data Are Available in Exercising Women and Athletes with Energy Deficiency and Menstrual Disturbances?

Research involving adolescent and young adult athletes and military Service Members provides evidence of associations between Triad components and an increased incidence of non-BSI musculoskeletal injury [36]; however, the strength and reliability of these associations varies. The relationship of Triad factors to musculoskeletal injury is important to identify and describe because if the Triad results in and is associated with the occurrence of these types of injuries, it should be described to raise the awareness of this factor and to stimulate further research. Most prevalent is the literature linking proxies for energy deficiency to musculoskeletal injury [139, 293, 326–329]. Studies of female high school athletes in the USA representing a broad range of sports, with injury incidence rates of 37–66% during a season, have demonstrated significant associations between higher EDE-Q scores and an increased injury risk, with higher average EDE-Q scores in injured compared with non-injured athletes and a two to four times greater odds of injury in athletes with elevated EDE-Q scores [139, 293, 326]. Associations between DE and musculoskeletal injury have also been demonstrated in female collegiate athletes. In a study of 57 athletes representing six university teams (basketball, volleyball, track, cross-country, triathlon, and soccer), 82% incurred a musculoskeletal injury during the season; based on a median split drive for thinness score on the Eating Disorder Inventory-3, women with high drive for thinness scores had more injuries compared with those with a low drive for thinness scores, and a drive for thinness was the strongest predictor of injury in a multivariate model [329]. In a mixed-sex group of collegiate university athletes (66% female), however, no significant associations between DE (Disordered Eating Screen for Athletes-6 score ≥ 3) and injury were observed, though injury (73.2%) and DE (33.9%) were prevalent [330]; however, results may have been confounded by the combined analysis of men and women. In another mixed-sex cross-sectional analysis that included US Marine Corps officers participating in a 6-month secondary training course, officers with elevated DE (EDE-Q Short) ≥ 15) had 1.2 times greater odds of injury than those without elevated DE [328]. However, results were sex specific such that, within female (12% of total sample, 11% with elevated DE and 26% reported a musculoskeletal injury) but not male officers, those with elevated EDE-Q Short scores had a 1.4 times greater odds of injury compared with those without elevated scores [328].

Evidence linking non-BSI musculoskeletal injury to menstrual dysfunction and/or low BMD is less consistent [139, 293, 326, 331, 332]. In two reports of female high school athletes (*n* = 249–311), athletes with menstrual irregularity (primary amenorrhea, secondary FHOA) did not have a significantly greater incidence of injuries compared to regularly menstruating athletes [293, 331]. Conversely, in 163 female high school athletes representing a variety of sports across eight interscholastic teams, in unadjusted models, FHOA in the past year was associated with a 2.9 times greater odds of injury and a BMD Z-score < − 1.0 was associated with a 3.6 times greater odds of injury; these risk factors remained significant in the multivariate models that included DE (EDE-Q score), menstrual status, and BMD [139]. A similar study of 89 high school runners assessed multivariate models that encompassed DE (EDE-Q score), menstrual dysfunction (primary amenorrhea or secondary FHOA), and low BMD (Z-score < − 1.0 or Z-score < − 2.0): a BMD Z-score < − 1.0 was associated with a 4.6 times greater odds of injury, while a BMD Z-score < − 2.0 was no longer associated with injury, but in this model, menstrual dysfunction was now associated with a 4.1 times greater odds of injury. Disordered eating was not associated with injury in either multivariate model [326]. Finally, one investigation has evaluated associations between Triad components and musculoskeletal injury in 116 collegiate athletes utilizing the CRA tool, reporting a greater proportion of athletes in the moderate-high risk group were injured (55%) compared with the low-risk group (29%) [332].

Although the mechanisms contributing to the negative effect of chronic energy deficiency on bone mass and BSI risk are well described, processes explaining the relationship among factors consistent with energy deficiency and non-BSI musculoskeletal injury are unknown. An insufficient intake of energy may also contribute to inadequate intake of protein and micronutrients involved in collagen formation and tissue repair [333]. As protein synthesis and tissue repair and formation require energy, energy deficiency may limit these processes and increase the likelihood for soft-tissue injury [334].

**Evidence-Based Statement:** Energy deficiency (as evidenced by ED/DE) is associated with a higher risk of musculoskeletal injuries. **Grade:** Level C.

**Evidence-Based Statement:** FHOA and low BMD may be associated with a higher risk of musculoskeletal injuries. **Grade:** Level C.

### What are the Cardiovascular Perturbations of Energy and Estrogen Deficiency?

Observational studies report cardiovascular perturbations in recreational and competitive Triad-affected female athletes with secondary amenorrhea [335–356], with some studies including athletes with primary amenorrhea [335, 340, 356], eumenorrheic anovulatory cycles (305, 306), and hormonal contraceptive users [335]. Altered vascular, autonomic, hemodynamic, and lipid profiles have been reported [335–356]. These are briefly described below.

#### Vascular

Compared with EAs, amenorrheic athletes demonstrate impaired brachial artery endothelium-dependent [339, 344, 351, 352, 355, 356] and endothelium-independent [344, 355] dilation, and lower regional blood flow [344, 348, 349] and arterial stiffness [343]. In eumenorrheic and amenorrheic athlete groups that include hormonal contraceptive users, vascular measures do not differ [335]. In oligomenorrheic athletes, endothelium-dependent dilation is similar [356] and lower [352], and endothelium-independent dilation is similar [352, 356] compared to EAs. Resumption of menses, hormonal contraceptive use, and supplementation with folic acid (10 mg/day for 4 weeks) reverse endothelial dysfunction in amenorrheic athletes [339, 351, 355].

#### Heart Rate and Heart Rate Variability

Most [343–346, 348, 353], but not all [335, 339, 351, 352, 354, 356], studies report a lower resting heart rate in amenorrheic (44–48 beats/min) compared with eumenorrheic athletes (54–57 beats/min) [343–346, 348, 353]. Oligomenorrheic and eumenorrheic athletes demonstrate similar resting heart rate [352, 356]. During simulated orthostatic stress, amenorrheic athletes demonstrate lower [345, 346] and similar [354] heart rates than/to eumenorrheic athletes. Similar baroreflex sensitivity, yet higher heart rate variability and parasympathetic modulation of heart rate have also been reported in amenorrheic versus eumenorrheic athletes, both at rest [346, 354] and during simulated orthostatic stress [346].

#### Blood Pressure

Amenorrheic athletes demonstrate lower [339, 343–346, 348] and similar [335, 351–353, 356] resting systolic blood pressure compared with eumenorrheic athletes, with values that range between 91 and103 mmHg compared with between 101 and 112 mmHg, respectively. Oligomenorrheic and eumenorrheic athletes demonstrate similar blood pressure [352, 356]. Despite lower blood pressure, amenorrheic athletes have elevated basal regional [344, 348, 357] and systemic [344] vascular resistance. During simulated orthostatic stress, muscle sympathetic nerve activity is augmented yet the anticipated activation of the renin-angiotensin system is not observed in amenorrheic compared with eumenorrheic athletes [345].

#### Lipid Profile

Lipid changes in amenorrheic, but not oligomenorrheic [352], athletes have been reported, with some [338, 341, 352] but not all [335–337, 342, 344, 350] studies demonstrating elevated levels of low- and high-density lipoprotein cholesterol, total cholesterol, and triglycerides [338, 341, 352] when compared with eumenorrheic athletes. In amenorrheic athletes, low-density lipoprotein cholesterol is associated inversely with endothelium-dependent dilation [352] and is potentially more susceptible to oxidation after exercise than in eumenorrheic athletes [336, 337, 352].

### What is Causing the Cardiovascular Perturbations in Triad-Affected Female Athletes?

Estrogen, exercise, and mild caloric restriction are independently associated with favorable effects on endothelial function [358–360], lipid profile [361–363], resting blood pressure and heart rate [361, 364–367], and heart rate variability [365, 367, 368]. Conversely, estrogen deficiency is associated with vascular dysfunction [358], altered blood pressure and heart rate regulation [366, 369], and impaired lipid metabolism [363]. Thus, cardiovascular perturbations in the amenorrheic athlete are likely the result of complex competing interactions between the effects of energy deficiency, estrogen deficiency, and exercise training.

**Evidence-Based Statement:** Amenorrheic athletes may demonstrate impaired endothelium- dependent and endothelium-independent dilation that may be accompanied by hemodynamic, autonomic, and lipid profile changes. **Grade:** Level B.

**Evidence-Based Statement:** Oligomenorrhea in athletes may be associated with impaired endothelium-dependent dilation. **Grade**: Level C.

## Summary and Recommendations of the 2025 Update on the Female Athlete Triad

This article has considered all types of evidence to generate expert opinions related to various components of the Triad. Particularly, data are now included from many RCTs and prospective studies. However, some cross-sectional data are also included.

As opportunities for girls and women in sport have greatly expanded throughout the world, the number of girls and women participating in sport continues to grow and the performance standards for girls and women are continuing to drive higher and higher standards of excellence and more demanding training paradigms. Despite the health benefits of exercise, the existence of the Triad remains a serious threat to the immediate and long-term health of female athletes. Prevalence rates of Triad outcomes remain high in adult and adolescent athletes [370]. Specifically, prevalence rates of 70% are reported for some groups of adolescent athletes [290–295], and as such, diligent surveillance, prevention, and treatment efforts continue to be of high priority. Since the Triad was first defined, the conditions comprising it, i.e., energy deficiency with or without EDs, menstrual disturbances, and poor bone health, have been, and still are, the most common and deleterious conditions facing female athletes who do not match energy intake to energy needs. While much research needs to be done, there is an equal need for Triad education and evidence-based policymaking as it concerns the prevention and treatment of Triad-related health issues. The Female and Male Athlete Triad Coalition is committed to supporting research, education, and improvements in clinical care, and notably, has recently partnered with the National Federation of High Schools in the USA to develop an online educational program to inform high school coaches about the Triad in girls/women and men.

### Building on the Evidence-Based Statements Presented in the Updated 2025 Consensus Statement, the Following Represent our Summary Recommendations

For the purpose of clarity and consistency in the field, Triad researchers and clinicians should adopt the following terminology referring to the energy continuum: energy status, energy deficiency, energy replete, and metabolic compensation. We have provided clear definitions and advocate for consistent use in all future Triad literature. In particular, we recommend the use of the term “energy deficiency” instead of the term “low EA” as it applies to its causal role in the induction of Triad outcomes. Given the poor reliability and difficulties with the assessment of EA, we recommend the term “low EA” be used as one of several indicators of energy deficiency.

Triad researchers and clinicians should recognize that metabolic compensation is a sign of energy deficiency and that assessments of energy deficiency should attempt to capture these adaptations. As such, the RMR ratio and RMR kcal/kg FFM and endocrine adaptations may be helpful for screening, diagnosis, and treatment monitoring of energetic status.

Triad researchers and clinicians should take into account the known differences in the rates of induction and reversal of each Triad outcome, for example, the rate of induction of metabolic perturbations resulting from energy deficiency is faster than the rate of reversal of metabolic perturbations during refeeding, and the rate of induction of menstrual cycle disturbances appears to be more rapid than the reversal of menstrual cycle disturbances to cycles that are healthy ovulatory successive cycles of normal length. In general, induction of Triad conditions occurs more rapidly than the reversal of Triad conditions.

We recommend reproductive recovery should encompass resumption of menses and/or increased menstrual frequency, *and* ovulatory cycles. Functional hypothalamic oligo*-*amenorrhea may co-exist with hyperandrogenism, and we recommend that hyperandrogenism be considered as a possible cause of the absence of menstrual recovery in athletes who do not resume menses despite addressing underlying energy deficiency.

The emergence of evidence supporting the roles of gynecological age and psychosocial stress as modifiers of the susceptibility of the HPO axis to energy deficiency in exercising women should alert researchers and clinicians to incorporate these factors into the care, study, and prevention of the Triad. Relevant considerations include:

The risk of Triad-related conditions is reduced with increasing gynecological age.

Greater psychosocial stress may modify the susceptibility of an exercising woman to develop menstrual disturbances related to the Triad.

Current evidence suggests that researchers and clinicians should be aware of differences in the presentation of the Triad between adults and adolescents. Regarding ED and DE, we recommend increased awareness of the diagnosis of atypical anorexia nervosa, a variant of the other specified feeding and other specified ED that may commonly present in certain athlete types, which does not require objectively low weight status. Triad researchers and clinicians should also consider the broad panoply of individual- and system-level factors that may contribute to risk for engagement in ED-related behaviors.

The emergence of evidence supporting the inclusion of BSIs in both the adult and adolescent Triad models should alert researchers and clinicians to incorporate Triad measurements into the overall study and prevention of BSIs. Emerging areas of concern in the science affecting female athletes include the associations of the Triad to both musculoskeletal injury and to cardiovascular factors.

In both adolescent and adult athletes, Triad-related factors such as lower BMI, DE behavior, delayed menarche, menstrual irregularity, and low BMD Z-scores may be risk factors for musculoskeletal injury. The long-term health consequences and clinical relevance of altered cardiovascular changes in exercising women with FHOA are currently unknown, caution is warranted when interpreting observations from cross-sectional studies and extrapolating them to long-term cardiovascular consequences in association with the Triad.

## Future Directions

With the increasing participation of girls and women in sport and exercise, an updated study that evaluates the prevalence of the Triad components is critically needed to capture the magnitude of Triad conditions in 2025 and such a study should capture both the individual and combined Triad components and include multiple ethnic and racial minorities.

Future research efforts should prioritize quantifying the prevalence and incidence of clinical eating pathology (across the weight spectrum, and at both the symptom and diagnostic level) among athletes, as well as individual- and system-level factors that may contribute to the risk for engagement in these ED-related behaviors. The knowledge gained will serve to inform and improve clinical screening, athlete-tailored treatment efforts, and allocation of resources.

Further prospective studies employing repeated measures of growth, BMI percentile, and bone mineral accumulation are needed in adolescent athletes. Future research should clarify and identify optimal nutritional intervention strategies required to restore complete reproductive recovery including the sustained recovery of ovulatory cycles of normal length. More research is needed to optimize strategies to recover bone secondarily to nutritional interventions alone and in combination with hormonal therapies. Prospective studies are needed to test whether psychosocial stressors are causally related to menstrual disturbances in exercising women independent of energy deficiency.

## Final Conclusions

Advances in research and clinical practice since the 2014 Female Athlete Consensus Statement [1–3] have continued to build out the evidence-based conceptual model of the Triad, improving its specificity, scalability, and accuracy. Although more research is needed, in this 2025 update, we have carefully considered how the Triad presents in adolescents and acknowledged the reduced susceptibility to menstrual disturbances with increasing gynecological age. More information regarding the impact of psychosocial stress and hyperandrogenism on menstrual cycles has been included. We have provided more context regarding the quantification of low EA, and new information on the issue of an EA threshold. New information regarding the reversibility of reproductive dysfunction and low BMD with refeeding is presented. Overall, progress has been made in our understanding of the underlying mechanisms and interrelatedness of all aspects of the Triad. The extent to which this new knowledge and new recommendations are incorporated into research and clinical practice will depend on the willingness of sports medicine practitioners, coaches, athletes, and other stakeholders to prioritize careful and evidence-based approaches over more broad-based non-Triad specific perspectives [13] (Tables 3 and 4). Last, a second paper, *2025 Update to the Female Athlete Triad Coalition Consensus Statement Part 2: Clinical Guidelines for* S*creening, Diagnosis, and Treatment and Recommendations for Clearance and Return to Play for the Adolescent and Adult Female Athlete Triad*, is also included as an update paper and provides updated clinical information and a revised CRA tool for diagnosis, treatment, and return to play for female athletes. Both 2025 update papers represent a consensus of the state of the science and recommendations for physicians, sports medicine practitioners, other healthcare providers, and researchers that was developed by the Female and Male Athlete Triad Coalition.

**Table 3:** Female Athlete Triad Adult Model: Evidence-Based Statements

*Scientific updates to the Energy Status Continuum*
**Evidence-Based Statement:** Research in untrained women who underwent a 3-month exercise intervention that included caloric restriction does not support the use of an absolute threshold of EA as a strategy to prevent menstrual disturbances; rather, there is individual variability in the level of EA below which menstrual disturbances are induced and there likely exists a dose–response continuum between energy status and menstrual function
*EVIDENCE GRADE:* Level A
**Evidence-Based Statement:** In non-laboratory settings, the measurement of EA is problematic because of low reliability in the assessment of its components and high day-to-day fluctuations
*EVIDENCE GRADE:* Level B
*Scientific updates on eating disorders and disordered eating*
**Evidence-Based Statement:** Although some aspects of sport participation are protective against negative mental health outcomes, female athletes may be at unique risk for the development of eating pathology, particularly when engaged in sports that promote a certain body type
*EVIDENCE GRADE:* Level B
**Evidence-Based Statement:** ED risk factors for female athletes include sport-specific (such as early sport specialization and coach/teammate influence) and general factors (including a perfectionistic temperament and stress)
*EVIDENCE GRADE:* Level C
*Scientific Updates to the Reproductive Function Continuum*
**Evidence-Based Statement:** Menstrual disturbances associated with reduced or suppressed ovarian steroid exposure are induced in young normal weight, ovulatory women with moderate weight loss of 3–4 kg and with a moderate energy deficit of 500–800 kcal/day over 2–3 months
*EVIDENCE GRADE:* Level A
**Evidence-Based Statement:** Total or free T_3_ can be used as a surrogate marker for energy status in female athletes with energy deficiency and resulting reproductive sequelae, and concentrations often normalize with improved energy status
*EVIDENCE GRADE:* Level B
**Evidence-Based Statement:** The risk of developing menstrual disturbances in association with energy deficiency declines as gynecological age increases
*EVIDENCE GRADE:* Level B
**Evidence-Based Statement:** Psychosocial factors should be considered as influencing the development of energy deficiency-associated menstrual disturbances
*EVIDENCE GRADE:* Level B
**Evidence-Based Statement:** The simple resumption of menses or an increase in menstrual frequency, while an important step in the recovery journey, does not always indicate complete reproductive recovery
*EVIDENCE GRADE:* Level A
**Evidence-Based Statement:** A healthy target for an exercising woman recovering from FHOA is the onset of menses followed by multiple consecutive cycles (we recommend greater than two cycles of < 36 days)
*EVIDENCE GRADE:* Level A
**Evidence-Based Statement**: Increases in estrogen exposure in exercising women recovering from FHOA are unlikely until three consecutive cycles of < 36 days are achieved
*EVIDENCE GRADE:* Level A
**Evidence-Based Statement:** An increase in energy intake between 300 and 600 kcal/day is often adequate to initiate reproductive recovery (i.e., increased frequency of menses) in FHOA exercising women
*EVIDENCE GRADE:* Level A
**Evidence-Based Statement:** The time course to reproductive recovery may be longer in women with greater duration of menstrual dysfunction
*EVIDENCE GRADE:* Level A
**Evidence-Based Statement:** Reproductive recovery with increased energy intake is more likely in FHOA exercising women with higher baseline fat mass
*EVIDENCE GRADE:* Level A
**Evidence-Based Statement:** Many athletes’ perceptions of amenorrhea and/or exercise-associated menstrual disturbances reflect a poor understanding and/or a denial of the severe health consequences of amenorrhea
*EVIDENCE GRADE:* Level C
*Scientific updates to the Bone Health Continuum*
**Evidence-Based Statement:** Athletes with FHOA demonstrate lower BMD, and impaired bone geometry, structure, and strength estimates compared with athletes with eumenorrhea and non-athletes, particularly at non-weight-bearing sites; energy deficiency has a greater impact on these parameters at weight-bearing sites
*EVIDENCE GRADE:* Level A
**Evidence-Based Statement:** Athletes with FHOA demonstrate alterations in bone turnover markers consistent with their estrogen and/or energy status
*EVIDENCE GRADE:* Level A
**Evidence-Based Statement:** Evidence of energy deficiency and FHOA are important determinants of BSI in athletes
*EVIDENCE GRADE:* Level A
**Evidence-Based Statement:** The risk of low BMD is higher in exercising girls and women as the number of Triad risk factors increases (cumulative risk)
*EVIDENCE GRADE:* Level A
**Evidence-Based Statement:** The risk of BSI is higher in exercising girls and women as the number of Triad risk factors increases (cumulative risk)
*EVIDENCE GRADE:* Level A
**Evidence-Based Statement:** Female athletes with a higher cumulative Triad risk have a greater risk for trabecular-rich BSI compared to cortical-rich BSI
*EVIDENCE GRADE:* Level B
**Emerging issues**
**Evidence-Based Statement:** Energy deficiency (as evidenced by ED/DE) is associated with a higher risk of musculoskeletal injuries
*EVIDENCE GRADE:* Level C
**Evidence-Based Statement:** FHOA and low BMD may be associated with a higher risk of musculoskeletal injurie
*EVIDENCE GRADE:* Level C
**Evidence-Based Statement:** Amenorrheic athletes may demonstrate impaired endothelium-dependent and endothelium-independent dilation that may be accompanied by hemodynamic, autonomic, and lipid profile changes
*EVIDENCE GRADE:* Level B
**Evidence-Based Statement:** Oligomenorrhea in athletes may be associated with impaired endothelium-dependent dilation
*EVIDENCE GRADE:* Level C

*BMD* bone mineral density, *BSI* bone stress injury, *DE* disordered eating, *EA* energy availability, *ED* eating disorder, *FHOA* functional hypothalamic oligo/amenorrhea

**Table 4:** Female Athlete Triad Adolescent Model: Evidence-Based Statements

Introducing an adolescent triad model
**Evidence-Based Statement:** The adolescent years are a critical time of life to optimize bone accrual and overall health
*EVIDENCE GRADE:* Level A
**Evidence-Based Statement:** We concur with defining primary amenorrhea as no menses by age 15 years, and secondary amenorrhea as the absence of menses for more than 90 days after 1–2 years post-menarche
*EVIDENCE GRADE:* Level A
**Evidence-Based Statement:** Energy deficiency and suboptimal nutrient intake in adolescent female athletes can result in delayed menarche, primary or secondary amenorrhea, low BMD, reduced bone accrual and low peak bone mass
*EVIDENCE GRADE:* Level A
**Evidence-Based Statement:** The prevalence of energy deficiency among adolescent female athletes is variable depending on the nature of sports activity
*EVIDENCE GRADE:* Level B
**Evidence-Based Statement:** Adolescent athletes engaged in aesthetic, weight-class, endurance, leanness, or non-weight-bearing sports are at increased risk for late age at menarche, primary or secondary amenorrhea, and low BMD
*EVIDENCE GRADE:* Level A
**Evidence-Based Statement:** Participation in aesthetic, weight class, endurance, and/or leanness sports may increase the risk of DE
*EVIDENCE GRADE:* Level B
**Evidence-Based Statement:** Adolescent athletes are at higher risk of menstrual irregularity compared with non-athletes
*EVIDENCE GRADE:* Level B
**Evidence-Based Statement:** In adolescents, eumenorrheic athletes or athletes participating in multi-directional impact-loading sports exhibit higher BMD values than non-athletes
*EVIDENCE GRADE:* Level A
**Evidence-Based Statement:** Adolescent athletes with FHOA are at risk for low BMD, particularly at estrogen-sensitive sites, with impaired bone geometry, structure, and strength estimates
*EVIDENCE GRADE:* Level A
**Evidence-Based Statement:** Low BMI, and/or clinically significant weight loss, and/or tracking below the weight trajectory on the CDC growth curve, may indicate energy deficiency and risk for FHOA, both of which are key risk factors for low BMD in female adolescent athletes
*EVIDENCE GRADE:* Level A
**Evidence-Based Statement:** Female adolescent athletes participating in cross-country, track and field, and gymnastics exhibit an elevated BSI risk affected by BMD, age at menarche, menstrual function, sport type, sports participation history, prior injury, and eating attitudes and behaviors
*EVIDENCE GRADE:* Level B
**Evidence-Based Statement:** While low BMD among female adolescent athletes appears possibly irreversible in the short term, normalization of weight and menstrual function of sufficient duration in some athletes may allow for catch-up bone mineralization and normalization of bone mass
*EVIDENCE GRADE:* Level C

*BMD* bone mineral density, *BMI* body mass index, *BSI* bone stress injury, *CDC* Centers for Disease Control and Prevention, *FHOA* functional hypothalamic oligo/amenorrhea
